# Membrane-Anchored and Sequence-Oriented Antiviral Activity of Fusion-Inhibitory Lipopeptides Derived from the SARS-CoV-2 Spike Glycoprotein S2 Subunit

**DOI:** 10.3390/v18060682

**Published:** 2026-06-18

**Authors:** Rosaria Arvia, Michael Quagliata, Andrea Di Santo, Maria Alfreda Stincarelli, Lorenzo Pacini, Anna Maria Papini, Paolo Rovero, Simone Giannecchini

**Affiliations:** 1 Department of Experimental and Clinical Medicine, University of Florence, Viale Morgagni 48, 50134 Florence, Italy; rosaria.arvia@unifi.it (R.A.); mariastincarelli@gmail.com (M.A.S.); 2 Interdepartmental Research Unit of Peptide and Protein Chemistry and Biology, Department of Chemistry “Ugo Schiff”, University of Florence, 50019 Sesto Fiorentino, Italy; michael.quagliata@unifi.it (M.Q.); l.pacini@unifi.it (L.P.); annamaria.papini@unifi.it (A.M.P.); 3 Interdepartmental Research Unit of Peptide and Protein Chemistry and Biology, Department of NeuroFarBa, University of Florence, 50019 Sesto Fiorentino, Italy; andrea.disanto@unifi.it

**Keywords:** SARS-CoV-2, spike S2 subunit, antiviral peptide, lipopeptide, PEG spacer

## Abstract

Background: SARS-CoV-2 fusion inhibitory peptides represent promising antiviral candidates. Recently, a 19-mer peptide (PN19)—designed in our laboratory to mimic the internal fusion peptide of the SARS-CoV-2 spike S2 subunit—demonstrated potent antiviral activity and stable conformational features. Objectives: To investigate how this antiviral activity depends on membrane interactions, we designed synthetic PN19 lipopeptide derivatives and evaluated their efficacy against SARS-CoV-2 replication. Methods: Lipopeptides were synthesized by conjugating cholesterol to either the N- or C-terminus of the PN19 peptide, utilizing a Gly/Ser pentapeptide (GSGSG) and/or various polyethylene glycol (PEG) spacers. Antiviral activity against SARS-CoV-2 variants was evaluated by plaque reduction assays, and cytotoxicity was assessed in Vero E6 cells. Results: The lipopeptides exhibited potent inhibitory activity at sub-micromolar concentrations. Compared to the unmodified PN19 peptide, antiviral efficacy was significantly enhanced by cholesterol conjugation at either terminus. Evaluation of six PN19 lipopeptides bearing the GSGSG sequence and different PEG spacers revealed that C-terminal cholesterol conjugation yielded higher antiviral activity than N-terminal derivatives. Furthermore, thirteen shorter PN19 lipopeptide derivatives (8–13-mers) confirmed this robust efficacy, which was most pronounced with C-terminal cholesterol conjugation and further enhanced by the spacers. Noteworthy, all tested PN19 lipopeptides displayed broad activity against multiple SARS-CoV-2 variants in the absence of cytotoxicity. Conclusions: Collectively, peptides conjugated with cholesterol at the C-terminus emerged as highly potent inhibitors of SARS-CoV-2, likely driven by enhanced peptide–membrane interactions. These findings warrant further investigation to fully elucidate the role of lipidation in the inhibitory mechanism, supporting the development of novel antiviral lipopeptides for SARS-CoV-2 therapy.

## 1. Introduction

Emerging and re-emerging outbreaks of novel coronaviruses, such as severe acute respiratory syndrome coronavirus 2 (SARS-CoV-2), represent a critical threat to global public health [1,2,3]. To date, available SARS-CoV-2 vaccines have exhibited limited long-term efficacy, while approved antivirals and therapeutic monoclonal antibody treatments have shown diminished effectiveness against evolving variants [1,4]. Consequently, the development of novel antiviral strategies is urgently warranted. Among the investigated options, peptides derived from the conserved domains of the Spike glycoprotein—designed to inhibit viral receptor binding or virus–cell fusion—are being extensively studied [5,6]. However, although the molecular process that drives the fusion of viral and host membranes is conserved across viruses, the rapid exposure of the target during this mechanism makes achieving effective antiviral activity extremely challenging [7]. Thus, new strategies are needed to increase peptide accessibility during this brief window of target exposure [6,8,9]. In several studies on antiviral fusion inhibitor peptides against SARS-CoV-2, mirroring strategies previously validated for HIV, peptides have been engineered for increased structural stability or conjugated to lipid moieties (principally cholesterol). Additionally, flexible spacers of varying lengths, such as polyethylene glycol (PEG), have been deployed to enhance antiviral potency [5,6,10,11,12,13]. The rationale underlying these strategies is to allow the cholesterol-conjugated peptide to act as an anchor, stably localizing within lipid membranes to interfere with the interaction between the viral spike protein and the host cell membrane receptor directly at the site of action [14,15,16,17,18]. This localization is driven by the natural tendency of cholesterol to partition into lipid rafts within both cellular and viral membranes, where the angiotensin-converting enzyme 2 (ACE2) receptor and the viral spike protein co-localize [15,19,20,21,22].

Recently, our laboratory identified a 19-mer peptide (PN19) derived from the SARS-CoV-2 spike S2 internal fusion peptide domain. PN19 demonstrated robust antiviral activity owing to favorable conformational features that enable it to target the S2 pre-membrane domain [23]. This inhibitory effect was mapped to a short segment containing three phenylalanine (Phe) residues sequentially separated by eight and nine amino acids, respectively [23]. Furthermore, this activity was time-dependent relative to infection, directly targeting a transient site exposed on the viral spike during viral-cell adsorption.

In the present study, we leveraged the membrane-anchoring propensity of cholesterol to synthesize a series of cholesterol-modified PN19 peptides and evaluate their antiviral efficacy. To optimize the spatial exposure of the conjugated PN19 peptide, we introduced either a short peptide spacer, a flexible PEG linker, or a combination of both between the active sequence and the cholesterol moiety [5,12]. This structural modulation was designed to position PN19 at varying distances from the membrane surface, thereby facilitating optimal interaction with its rapidly exposed target to achieve superior potency. Additionally, we evaluated truncated derivatives of PN19 conjugated with cholesterol and these spacers to investigate how these modifications influence the functional contribution of the core sequence’s minimal active residues.

## 2. Materials and Methods

### 2.1. Cells and Viruses

The cell lines used were Vero E6 (CRL-1586, ATCC, Rockville, MD, USA) cultivated using Dulbecco’s Modified Eagle’s Medium (DMEM) supplemented with 10% fetal bovine serum (FBS). The viruses used were SARS-CoV-2 isolates (SCV2/Fi/3/22 corresponding to the early pandemic Pango lineage B.1, SCV2/Fi/2/21 corresponding to Delta-like pango lineage B.1.617.2, and SCV2/Fi/1/22 corresponding to omicron-like pango lineage BA.1). All viruses were grown on Vero E6 cells, titrated by the plaque method, and stored at −80 °C until used.

### 2.2. Synthesis of 2-Cholesteryloxy-2-oxoethyl-bromide

2-Cholesteryloxy-2-oxoethyl-bromide was synthesized by Steglich esterification using bromo acetic acid. In a 50 mL round bottom flask, cholesterol (500 mg, 1.3 mmol, 1 eq), DCC (400 mg, 2.0 mmol, 1.5 eq) and DMAP (10 mg, 0.08 mmol, 0.06 eq) were dissolved in DCM (10 mL). Bromoacetic acid (200 mg, 1.4 mmol, 1.1 eq) was added and the solution was stirred at room temperature overnight, when TLC indicated appearance of a new less polar spot: R*_f_* = 0.70 (1:19 EtOAc/petroleum ether, visualized with KMnO_4_). The suspension was filtered and the solution was concentrated until 1/5 of the initial volume. The solution was placed at −20 °C for 2 h and the resulting suspension was filtered. Finally, the organic phase was removed under vacuum to afford cholest-5-en-3-yl bromoacetate as a white powder (500 mg, 76%). ^1^H NMR (200 MHz, CDCl_3_) δ (ppm) 5.36 (br, 1H), 4.61–4.65 (m, 1H), 3.57 (s, 2H), 2.34 (m, 2H), 2.05–0.80 (m, 38H), 0.67 (s, 3H). Data are in agreement with the literature.

### 2.3. Synthesis and Purification of Cys-peptide Precursors

The Cys-peptide precursors were synthesized by Induction-assisted Solid-Phase Peptide Synthesis (I-SPPS) following the Fmoc/*t*Bu orthogonal protection strategy, using the PurePep™Chorus™ automated peptide synthesizer (Gyros Protein Technologies, Uppsala, Sweden). Tentagel^®^ S RAM resin was used (loading 0.23 mmol/g). The following L-Fmoc-amino acids were used: Fmoc-Ala-OH, Fmoc-Cys(Trt)-OH, Fmoc-Phe-OH, Fmoc-Gly-OH, Fmoc-Leu-OH, Fmoc-Met-OH, Fmoc-Pro-OH, Fmoc-Gln(Trt)-OH, Fmoc-Arg(Pbf)-OH, Fmoc-Ser(*t*Bu)-OH, Fmoc-Tyr(*t*Bu)-OH, Fmoc-NH-PEG_6_-OH (CAS: 882847-34-9) and Fmoc-NH-PEG_12_-OH (CAS: 756526-01-9). Fmoc deprotections were performed with a solution of 20% piperidine in DMF for 60 s at 363 K. Peptide assembly was performed by repeating the SPPS standard coupling cycle for each amino acid, using Fmoc-protected amino acids (500 mol%), OxymaPure^®^ (5 eq), and DIC (5 eq) dissolved in DMF for 120 s at 363 K. The washing steps were performed using a mixture of 8:2 EtOAc/DMSO (*v*:*v*). All the Fmoc-Phe-OH were coupled twice. Fmoc-NH-PEG_6_-OH and Fmoc-NH-PEG_12_-OH were coupled manually (2 eq), using HATU (1.9 eq) and DIEA (3 eq) at r.t for 1 h. The *N*-acetylation was performed using a solution of 10% Ac_2_O in DMF for 4 min at 40 °C. Final cleavage and side-chain deprotections were performed using a mixture of TFA/TIS/H_2_O/EDT (94:3:1.5:1.5, *v*:*v*:*v*:*v*) at r.t. After 3 h the resin was filtered off. The peptides were precipitated with a cold mixture 1:1 Et_2_O/hexanes, centrifuged, and lyophilized. The crude Cys-precursor peptides were purified by semipreparative RP-HPLC on a Waters instrument (Separation Module 2695, detector diode array 2996) using a Sepax Bio-C18 column (Sepax Technologies, Newark, DE, USA) (5 μm × 250 × 10 mm), at 4 mL/min with solvent systems A (0.1% TFA in H_2_O) and B (0.1% TFA in ACN) using a gradient 30–90% B in A over 30 min.

### 2.4. Cholesterol Conjugation on Thiol Side Chain

**Figure 1:**
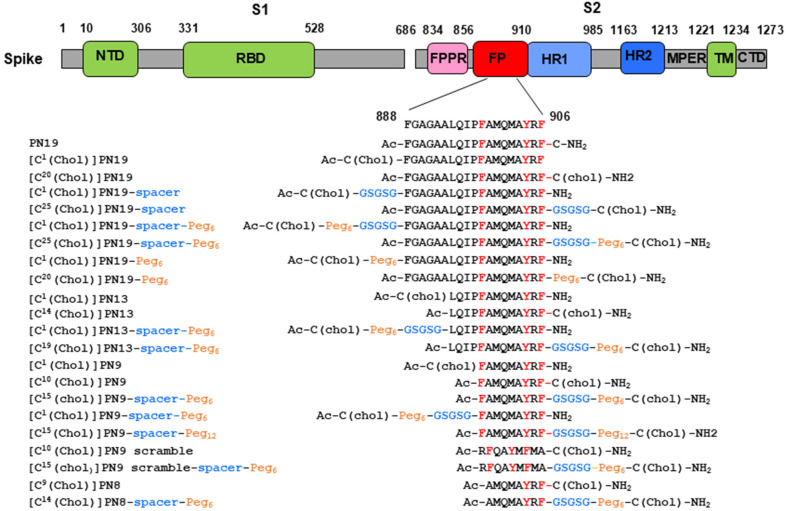
Schematic representation of the Spike glycoprotein subunits of SARS-CoV-2 and the localization of peptides used in the study. FPPR, fusion peptide proximal region. FP, fusion peptide (comprising the internal fusion peptide). HR1, heptad repeat 1. HR2, heptad repeat 2. MPER, membrane proximal region. TM, transmembrane region. Cyto, cytoplasmic region. Chol, cholesterol residue. GSGSG, a short pentapeptide sequence (Gly-Ser-Gly-Ser-Gly) used as spacer, is highlighted in blue. Peg_6,12_, polyethylene glycol spacer composed of 6 or 12 monomers is highlighted in orange. PN19 functional amino acid residues are highlighted in red [23].

The cholesterol was conjugated on the Cys side-chain exploiting a modified protocol in the literature [24]. In a 1.5 mL Eppendorf, purified Cys-precursor peptide (3.73 μmol, 1 eq) was dissolved in DMSO (850 μL) and treated with a solution of 2-Cholesteryloxy-2-oxoethyl-bromide (2.3 mg, 4.5 μmol, 1.2 eq) in THF (150 μL). DIEA (7.5 mg, 58 μmol, 15.5 eq) was added to the mixture and the solution was stirred at room temperature for 1 h, when HPLC indicated complete disappearance of the starting material. The solution was diluted with a mixture of 3:1 H_2_O/*t*BuOH (15 mL) and lyophilized. The white powder was triturated with petroleum ether (3 × 1 mL), and the residue was redissolved in a mixture of 3:1 H_2_O/*t*BuOH (10 mL) and lyophilized, obtaining the pure cholesteryl-conjugated peptide. The localization of PN19 peptide and lipidated derivatives used in the study are reported in Figure 1. Full analytical data (HPLC and MS) of the cholesteryl peptides are reported in the Supplementary Materials (Table S1, Figures S1–S42).

### 2.5. Inhibition of SARS-CoV-2 Infection

Vero E6 cells (2.5 × 10^5^ cells/well) were seeded overnight in 3 mL of growth medium in six-well plates at 37 °C with 5% CO_2_. SARS-CoV-2 of used at multiplicity of infection (MOI) of 0.01. Peptide powder was dissolved in dimethyl sulfoxide (DMSO). Virus was mixed 1:1 with 10-fold serial dilutions (final peptide concentrations of 100, 10, 1, and 0.1 μM in DMSO solution) of the peptides in 0.3 mL. After incubating this mixture for 1 h at 37 °C, it was inoculated onto the host cell monolayers for 1 h at 37 °C with 5% CO_2_. Control wells received virus mixed with DMSO solution alone. After incubation, the cells were washed with 1× PBS before receiving a 0.5% SeaPlaque™ agarose overlay in serum-free propagation medium. To optimize cell entry, the monolayer was supplemented with 2 μg/mL of L-1-tosylamido-2-phenylethyl chloromethyl ketone-treated trypsin (Sigma-Aldrich, St. Louis, MO, USA). Following 3 days of incubation, the monolayers were fixed with methanol, stained with 0.1% crystal violet, and scored for plaque-forming units (PFUs). Antiviral efficacy was calculated as the percentage of plaque reduction relative to the DMSO viral control. Finally, fifty percent inhibitory concentrations (IC_50_) were determined via Microsoft Excel’s predicted exponential growth curve function.

### 2.6. Cell Cytotoxicity Assay

Vero E6 cells (10^4^ cells/well in flat-bottom 96-well culture plate) were seeded overnight. Upon reaching confluence, the cell monolayers underwent twice 1xPBS wash and treated with 100 μL of DMEM alone or DMEM containing the appropriate concentrations of the peptides. The final peptide concentration added to the cells ranged from 0.1 to 100 μM in DMSO solution. After incubation at 37 °C in a CO_2_ for 72 h, cell viability was assessed using a 3-(4,5-dimethylthazolk-2-yl)-2,5-diphenyl tetrazolium bromide (MTT) kit (Roche, Milan, Italy) according to the supplier’s instructions. Cytotoxicity was calculated by dividing the average optical density of treated samples by the average of the samples in the presence of DMSO solution alone.

### 2.7. Statistical Analysis

Data were analyzed using a two-tailed Student’s *t*-test. All data represent three independent experiments, and values represent the mean ± standard deviation (SD), with *p* < 0.05 considered statistically significant.

## 3. Results

### 3.1. Effect of Pre-Incubation Time on Antiviral Activity of PN19 Lipopeptide

**Figure 2:**
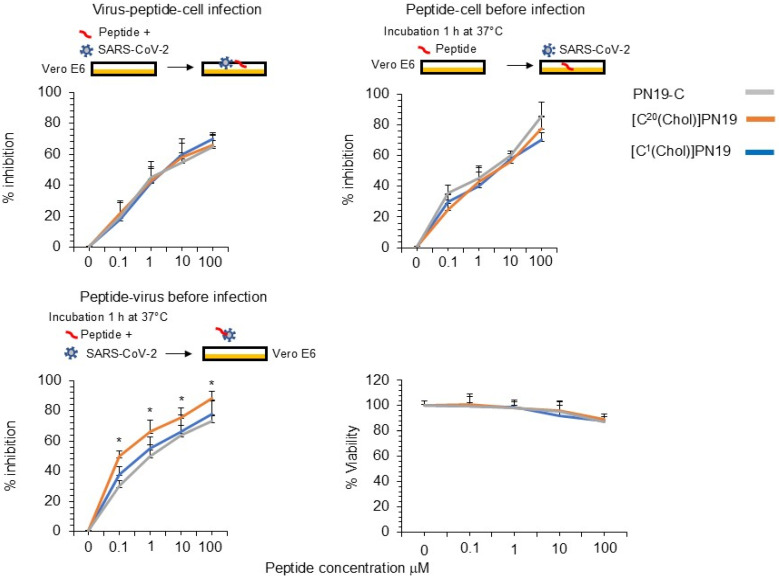
Inhibitory activity and cytotoxicity of peptide PN19 and lipo-derivatives against SARS-CoV-2 Vero E6 cell infection. SARS-CoV-2 infection of Vero E6 cells at MOI of 0.01 in presence of the indicated concentration of PN19 peptide and its lipopeptide derivatives [C^20^(Chol)]PN19 and [C^1^(Chol)]PN19 was evaluated with the viral plaque reduction assay (PRA) in three different conditions. Specifically, inhibitory activity was assessed by incubating peptides for 1 h at 37 °C with either virus (peptide-virus) or the cells (peptide-cells) before infection or by adding the peptide directly at the time of virus infection (virus–peptide-infection, standard procedure). Cell viability of Vero E6 cells, in the absence of viral infection, was determined via the MTT assay using same experimental condition used in PRA. The values shown are means ± standard deviation (SD) of three independent experiments. * *p* < 0.05, Student’s *t*-test.

Considering the time-dependent activity of PN19 and its target mechanism as previously reported [23], we investigated whether anchoring the PN19 lipopeptide derivative to the viral membrane could enhance its activity (Figure 1). First, we evaluated the antiviral activity of the PN19 lipopeptides when added directly at the onset of infection—the standard condition established in our previous study. This revealed that the inhibitory activity of PN19 was preserved following cholesterol modification at either the N- or C-terminus, yielding comparable efficacy to the unmodified PN19 peptide (Figure 2, Table 1).

Importantly, under the experimental conditions tested, neither PN19 nor its two cholesterol derivatives exerted a significant reduction in cell viability (Table 1). This result confirms that PN19 exerts its antiviral activity principally at the virus–cell adsorption site and that its cholesterol conjugation increases its effectiveness.

Next, we varied the incubation conditions of the PN19 lipopeptides relative to the time of viral infection. Pre-incubation of the PN19 lipopeptides with the virus prior to infection—but not pre-incubation with the cells—enhanced the inhibitory activity into the sub-micromolar range (Table 1). Importantly, under the experimental conditions tested, neither PN19 nor its two cholesterol derivatives induced a significant reduction in cell viability (Table 1). These results confirmed that PN19 exerts its antiviral activity principally during virus–cell adsorption, and that cholesterol conjugation substantially increases its effectiveness.

### 3.2. Effect of Different Spacer on Antiviral Activity of PN19 Lipopeptide

Subsequently, we evaluated the antiviral activity of the PN19 lipopeptide derivatives by pre-incubating the compounds with the virus prior to infection. To modify the positioning of the PN19 lipopeptide relative to the viral membrane, we synthesized six derivatives incorporating either a GSGSG peptide spacer, PEG linkers of varying lengths, or combinations of both. Among these newly synthesized compounds, introducing a GSGSG spacer combined with PEG_6_ to the N-terminal cholesterol derivative partially reduced antiviral activity, yielding a 50% inhibitory concentration (IC_50_) between 0.98 and 3.70 μM compared to the spacer-free N-terminal cholesterol control (Table 2). Conversely, the PN19 derivative bearing the C-terminal cholesterol modification alongside the GSGSG spacer and PEG_6_ exhibited robust antiviral activity, with an IC_50_ ranging from 0.04 to 0.7 μM. This represents a significant increase in potency compared to its counterpart with cholesterol alone (Table 2).

These results confirmed that, regardless of the presence or type of spacer utilized, C-terminal cholesterol modification of PN19 consistently yielded superior inhibitory activity compared to N-terminal modification. Again, none of the selected lipidated PN19 derivatives caused a significant reduction in cell viability (Table 2).

### 3.3. Antiviral Activity of Truncated PN19 Lipopeptide Derivatives

We next sought to investigate whether shorter fragments of the PN19 core sequence could retain antiviral efficacy. To this end, we synthesized a series of 13 lipopeptide derivatives (8–13-mers) of the PN19 peptide that replicate the conserved antiviral motifs identified in our previous study (Table 3).

Consistent with our previous observations, robust antiviral activity was predominantly achieved via C-terminal cholesterol modification, even among the shorter PN19 derivatives. Additionally, the influence of the spacer was length-dependent, with lipopeptide derivatives incorporating shorter PEG linkers exhibiting the highest potency (PEG_6_ vs. PEG_12_; Table 3). Notably, the complete loss of activity observed for the scrambled PN9 lipopeptide confirmed the necessity of the specific primary sequence. This sequence is characterized by a central phenylalanine (Phe) residue and a C-terminal tyrosine (Tyr) residue, both of which are crucial for the robust antiviral activity described in our previous study [23]. All antiviral lipopeptide derivatives demonstrated efficacy in the absence of cytotoxicity (Table 3).

**Figure 3:**
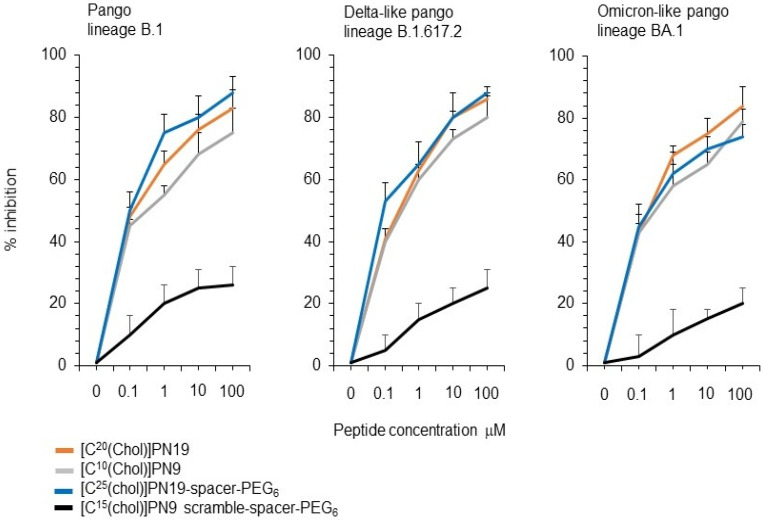
Inhibitory activity of selected small PN19 lipopeptide derivatives against SARS-CoV-2 variants Vero E6 cell infection. SARS-CoV-2 selected variants infection of Vero E6 cells at MOI of 0.01 in presence of the indicated concentration of PN19 lipopeptide derivatives was assayed with the viral plaque reduction assay in three different conditions. Lipopeptide control with the same length but with scrambled sequence was also used. The values shown are means + standard deviation (SD) of three independent experiments.

Finally, PN19 and the shorter PN9 lipopeptide derivatives were evaluated against different SARS-CoV-2 variants. As shown in Figure 3, all lipopeptide derivatives exerted efficient inhibitory activity across the variants tested (for all three SARS-CoV-2 variants, IC_50_ values ranged from 0.06 to 0.34 μM). Conversely, a lipopeptide with a spacer of the same length, but with a scrambled sequence, exhibited an impaired inhibitory activity (IC_50_ >100 μM for all three SARS-CoV-2 variants).

## 4. Discussion

In this study, the 19-mer peptide (PN19)—which reproduces the SARS-CoV-2 spike S2 fusion peptide sequence and exerts antiviral activity by targeting the S2 pre-membrane domain [23]—was modified to enhance its efficacy within the viral membrane microenvironment. To accomplish this, cholesterol was conjugated to either the N- or C-terminus of the sequence, a strategy designed to increase the local availability of the PN19 peptide during virus–cell fusion.

This was used in the light of cholesterol’s propensity to associate with lipid raft membrane domains, where virus–cell fusion occurs [17,18,25]. Additionally, to explore different spatial features of PN19, various spacer types and polyethylene glycol (PEG) lengths were introduced.

First, the results demonstrated that addition of cholesterol to the C-terminus of the PN19 peptide generated potent antiviral activity. This efficacy was particularly pronounced when the PN19 lipopeptide was incubated with the virus prior to cellular infection. Additionally, truncated derivatives of the PN19 lipopeptides confirmed the critical roles of the central Phe and C-terminal Tyr residues, aligning with previous findings [23]. We hypothesize that cholesterol modification preserves the core structure of PN19 while anchoring the peptide stably near the spike glycoprotein membrane domain, thereby improving its antiviral potency.

The superior activity observed when the PN19 lipopeptide was pre-incubated with the virus, rather than the cells, suggests that the peptide is already positioned for spike binding before the molecular events of virus–cell interaction and fusion initiate [26,27,28,29]. Furthermore, the high efficacy of C-terminal cholesterol addition compared to N-terminal addition indicates that the orientation of the PN19 sequence is critical for interacting with the target region at the membrane fusion site.

We previously suggested that the potential target of PN19 is the membrane-proximal external region (MPER) of the S2 spike subunit [23]. Recent studies have also proposed a fusion peptide (FP) interaction within this internal region, mediated by membrane cholesterol [30]. Specifically, the viral FP membrane-spanning helix is reported to undergo a sharp U-turn within the lipid bilayer, positioning its internal residues adjacent to the MPER domain in various orientations post-fusion [30]. Therefore, the PN19 lipopeptide may be more effective against its target region during a pre-fusion state when oriented by a C-terminal rather than an N-terminal cholesterol addition. This hypothesis is supported by literature showing that N- versus C-terminal cholesterol modifications on fusion-inhibitory peptides yield varying antiviral activities due to distinct orientations [24,31]. Alternatively, these differing orientations may alter the primary target of PN19.

A second key finding relates to the different activities of PN19 lipopeptides when using spacers of different lengths. While the GSGSG spacer and the PEG_6_ linker exhibited comparable efficacy, increasing the number of PEG monomers from 6 to 12 significantly reduced inhibitory activity. Extending the length of the GSGSG and PEG spacers likely masked the active peptide sequence, positioning it too far from the target or hindering peptide adsorption into the target membrane site. Notably, a similar decrease in antiviral efficacy with increasing PEG length has been documented for other inhibitory peptides [14].

Substantial evidence highlights viral entry as a primary target for developing therapeutics to counteract viral transmission [5,6]. While designing spike-glycoprotein-derived peptides to block viral fusion is well-documented across several viruses [5,6,7], major challenges remain. Specifically, selecting a highly conserved target region, ensuring target accessibility during fusion, and accounting for the narrow, time-dependent window of fusion intermediate availability remain principal concerns in developing effective antiviral compounds [26,27,28,29,30,31,32].

Notably, a lipidated peptide derived from the HR2 domain of the S2 subunit was shown to prevent viral transmission in vivo [14]. This efficacy is driven by the lipopeptide’s ability to adsorb onto the host cell membrane, thereby blocking SARS-CoV-2 entry [14]. In contrast, our lipopeptide, PN19—which is derived from the fusion peptide domain of the S2 subunit and targets a distinct viral region—exhibited reduced antiviral effectiveness upon cell membrane adsorption. Consequently, these findings make it challenging at present to propose PN19 lipopeptides as an in vivo preventative therapy for SARS-CoV-2. However, the potent inhibitory activity demonstrated by PN19 lipopeptides during viral pre-incubation highlights them as highly promising candidates that warrant further investigation for alternative therapeutic strategies. Future approaches could leverage alternative lipidation formulations to enhance specific interactions with the viral membrane, or utilize biodegradable polymer or hydrogel matrices to encapsulate the PN19 lipopeptide for sustained release. These delivery systems could prevent premature cellular adsorption, thereby preserving and enhancing its antiviral activity [33].

For SARS-CoV-2, as documented for HIV and other enveloped viruses [25,31,34,35], the predominant engineering strategy involves identifying viral spike protein sequences critical to the fusion process and introducing molecular modifications to stabilize the secondary peptide structure [6]. Additionally, adding a lipophilic tail to these candidates has emerged as a robust method to optimize peptide bioavailability at the target site during the narrow window of fusion intermediate accessibility [25,26].

**Figure 4:**
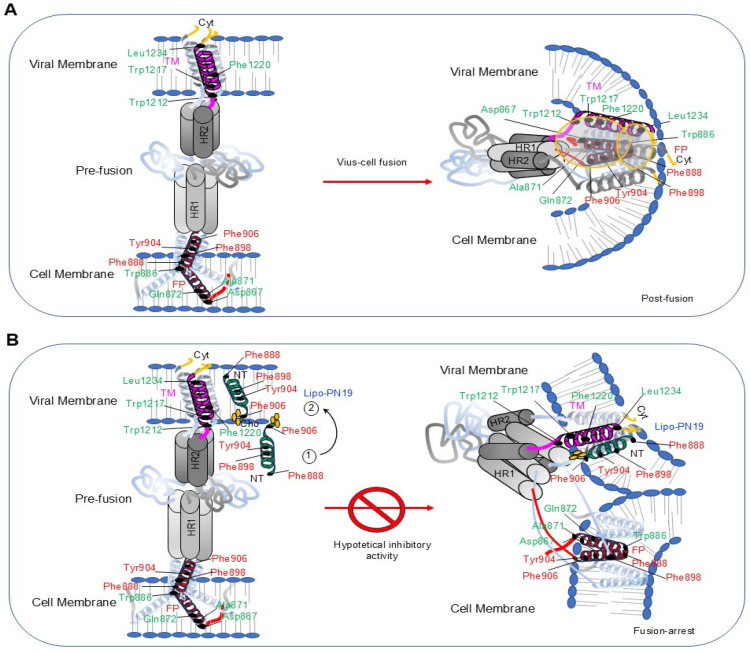
Proposed mechanism of action of PN19 lipopeptide based on the post-fusion spike structural model. (**A**) Schematic representation of the trimeric S2 subunits before and after viral–cell fusion, highlighting the interaction between the spike transmembrane region (TM) and the fusion peptide (FP); key residues are indicated within a single protomer, as previously revealed by cryo-electron microscopy [30]. (**B**) Schematic representation of the potential functional-interaction of the C-terminal cholesterol-modified PN19 lipidated peptide within the viral membrane (moving from a position reported in point 1 to position reported in point 2) adjacent to the spike transmembrane region TM. The potential inhibitory mechanism during fusion event is reported. Amino acid residues involved in TM and FP structural contact identified in previous study [29] (green: amino acids 867, 871, 872, 886, 1212, 1217, 1220, 1234) and those playing a key role in inhibitory activity of PN19 [23] (red: amino acids 888, 898, 904, 906) are highlighted within a single protomer. The yellow shaded circle enclosed the key interacting residues of the TM and FP structural domains [30]. TM, transmembrane; Cyt, cytosolic domain; FP, fusion peptide.

One limitation of this study is the lack of direct experimental data regarding the structural preferences of PN19, as well as its precise binding affinities to membranes in the absence and presence of cholesterol. However, the conformation of PN19, its key residues responsible for antiviral activity targeting the MPER region, and its behavior within membrane environments have been thoroughly documented in prior studies [23,36,37]. Furthermore, the membrane-partitioning propensity of cholesterol is a well-established feature leveraged in lapidated-peptide antiviral strategies [13,19,20,21,25,38]. Taking these data into consideration, a hypothetical mechanism of action—accounting for cholesterol membrane localization and the orientation-dependent activity of PN19—is proposed and illustrated in Figure 4, though it remains to be validated experimentally.

Collectively, our findings indicate that C-terminally cholesterol-conjugated peptides are potent inhibitors of SARS-CoV-2, confirming the efficacy of this membrane-anchoring antiviral approach. Further molecular investigations into the precise mechanism of action of these cholesterol-linked fusion peptides are warranted to guide the design of novel therapeutics for next-generation SARS-CoV-2 antiviral regimens.

## Figures and Tables

**Table 1:** Effect of cholesterol at N- or C-terminal on the antiviral activity and cell cytotoxicity of the PN19 peptides.

Peptide	IC_50_ (Mean ± SD) μM			CC_50_ (Mean ± SD) μM
	Standard	1 h Peptide-Virus	1 h Peptide-Cells	
[C^1^(Chol)]PN19	2.4 ± 1.04	0.18 ± 0.20	5.2 ± 1.10	>100
[C^20^(Chol)]PN19	3.4 ± 3.18	0.07 ± 0.31	2.8 ± 0.76	>100
PN19	0.9 ± 0.50	0.49 ± 0.15	2.4 ± 1.00	>100

IC_50_, 50 percent inhibitory concentration; CC_50_, 50 percent cytotoxicity concentration; C^1^(Chol), PN19 sequence amino terminal cholesterol addition; PN19 sequence C^20^(Chol), carboxy terminal cholesterol addition. Values obtained in two to three independent assays.

**Table 2:** Antiviral activity and cytotoxicity of PN19 lipopeptide derivatives.

Peptide	IC_50_ (Mean ± SD) μM	CC_50_ (Mean ± SD) μM
[C^1^(chol)]PN19-spacer	0.98 ± 0.12	>100
[C^25^(chol)]PN19-spacer	0.74 ± 0.34	>100
[C^1^(chol)]PN19-spacer-PEG_6_	1.75 ± 0.71	>100
[C^25^(chol)]PN19-spacer-PEG_6_	0.04 ± 0.02	>100
[C^1^(chol)]PN19-PEG_6_	3.70 ± 1.01	>100
[C^20^(chol)]PN19-PEG_6_	0.14 ± 0.12	>100
[C^1^ (chol)]PN19	0.38 ± 0.11	>100
[C^20^(chol)]PN19	0.08 ± 0.04	>100

IC_50_, 50 percent inhibitory concentration; CC_50_, 50 percent cytotoxicity concentration; C^X^(Chol), PN19 sequence amino terminal cholesterol addition; PN19 sequence C^X^(Chol), carboxy terminal cholesterol addition. Values obtained in two to three independent assays.

**Table 3:** Antiviral activity and cytotoxicity of PN19 shorts lipopeptide derivatives.

Peptide	IC_50_ (Mean ± SD) μM	CC_50_ (Mean ± SD) μM
[C^1^(Chol)]PN13	1.20 ± 1.04	>100
[C^14^(Chol)]PN13	0.28 ± 0.31	>100
[C^1^(Chol)]PN9	3.44 ± 0.90	>100
[C^10^(Chol)]PN9	0.27 ± 0.31	>100
[C^9^(Chol)]PN8	15.02 ± 3.04	>100
[C^10^(Chol)]PN9 scramble	>100	>100
[C^1^(chol)]PN13-spacer-PEG_6_	21.14 ± 10.12	>100
[C^19^(Chol)]PN13-spacer-PEG_6_	0.37 ± 0.31	>100
[C^1^(Chol)]PN9-spacer-PEG_6_	16.08 ± 8.04	>100
[C^15^(Chol)]PN9-spacer-PEG_6_	0.14 ± 0.11	>100
[C^14^(Chol)]PN8-spacer-PEG_6_	6.03 ± 3.05	>100
[C^15^(Chol)]PN9-spacer-PEG_12_	17.20 ± 6.07	>100
[C^15^(chol)]PN9 scramble-spacer-PEG_6_	>100	>100

IC_50_, 50 percent inhibitory concentration; CC_50_, 50 percent cytotoxicity concentration. C^1^(Chol), PN19 sequence amino terminal cholesterol addition; PN19 sequence C^X^(Chol), carboxy terminal cholesterol addition. Values obtained in two to three independent assays.

## Data Availability

All data generated or analyzed during this study are included in this published article. Further inquiries can be directed to the corresponding authors.

## Supplementary Materials

The following supporting information can be downloaded at https://www.mdpi.com/article/10.3390/v18060682/s1: The analytical data, the chromatograms, and mass spectrometry spectra are reported in Table S1 and Figures S1–S42.

## Abbreviations

SARS-CoV-2	severe acute respiratory syndrome coronavirus 2
ACE2	angiotensin-converting enzyme 2
PEG	poly(ethylene glycol)
DMEM	dulbecco’s modified eagle’s medium
FBS	fetal bovine serum
MOI	multiplicity of infection
DMSO	dimethyl sulfoxide
PRA	plaque reduction assay
IC_50_	50 percent inhibitory concentration
MTT	4,5-dimethylthazolk-2-yl)-2,5-diphenyl tetrazolium bromide
SD	standard deviation
Cho	cholesterol
CC_50_	50 percent cytotoxicity concentration
