# Supplementary material for: Membrane-Anchored and Sequence-Oriented Antiviral Activity of Fusion-Inhibitory Lipopeptides Derived from the SARS-CoV-2 Spike Glycoprotein S2 Subunit
Source: Viruses. 2026 Jun 18;18(6):682. doi: 10.3390/v18060682 (PMC13307882; doi:10.3390/v18060682)
Supplement: Supplementary file 1 [file viruses-18-00682-s001.zip › viruses-4368327-supplementary.pdf]

Characterization of cholesteryl-conjugated peptides

Analytical characterization of the cholesteryl-conjugated peptides was performed RP-HPLC Alliance Chromatography system (Waters, Milford Massachusetts, USA) coupled to a single quadrupole ESI-MS (Waters® ZQ Detector, Waters Milford, MA, USA) supplied with a Kromasil C4 (5 µm × 4.6 × 150 mm) column at 308 K, at 1 mL/min using solvent systems A (0.1% TFA in H_2O) and B (0.1% TFA in ACN). The analytical data, the chromatograms, and mass spectrometry spectra are reported in **Table S1** and **Figures S1-S42.**

Table S1: Analytical characterization of the cholesteryl-conjugated peptides. Eluents: 0.1% (*v*/*v*) TFA in H_2O (A) and 0.1% (*v*/*v*) TFA in ACN (B), λ 215 nm. Gradient: [a]50 - 100% B or [b]70 - 100% B in A over 10 min; [c]calculated as the ratio of obtained mass to theoretical mass. ESI-MS: detected as [d][M + 2H]$^{2+}$, [e][M + 3H]$^{3+}$, [f][M + H]$^{+}$.

Peptide	*HPLC R_t (min)[a]*	*HPLC purity (%)*	*Yield[c] (%)*	*ESI-MS (m/z) found[d] (calcd)*
**[C^{1}(chol)]PN19**	6.08[b]	91	23	1332.3 (1332.2)
**[C^{20}(chol)]PN19**	6.25[b]	91	24	1332.1 (1332.2)
**[C^{1}(chol)]PN19-spacer**	7.35	94	27	1004.1 (1004.3)[e]
**[C^{25}(chol)]PN19-spacer**	7.38	91	22	1004.1 (1004.3)[e]
**[C^{1}(chol)]PN19-PEG$_6$**	8.25	95	26	1000.9 (1000.7)[e]
**[C^{20}(chol)]PN19-PEG$_6$**	8.05	91	19	1000.8 (1000.7)[e]
**[C^{1}(chol)]PN19-spacer-PEG$_6$**	7.35	95	25	1116.0 (1116.2)[e]
**[C^{25}(chol)]PN19-spacer-PEG$_6$**	7.38	91	18	1116.0 (1116.2)[e]
**[C^{1}(chol)]PN13**	9.18	90	23	1095.3 (1095.2)
**[C^{14}(chol)]PN13**	9.22	93	25	1095.3 (1095.2)
**[C^{1}(chol)]PN9**	9.08	94	19	869.3 (869.2)
**[C^{10}(chol)]PN9**	8.92	93	21	869.3 (869.2)
**[C^{1}(chol)]PN13-spacer-PEG$_6$**	7.40	95	25	957.8 (957.6)[e]
**[C^{19}(chol)]PN13-spacer-PEG$_6$**	7.58	95	24	957.7 (957.7)[e]
**[C^{1}(chol)]PN9-spacer-PEG$_6$**	7.42	96	17	1210.0 (1209.7)
**[C^{15}(chol)]PN9-spacer-PEG$_6$**	7.48	96	20	1210.0 (1209.7)
**[C^{9}(chol)]PN8**	8.38	94	19	1590.3 (1590.1)[f]
**[C^{14}(chol)]PN8-spacer-PEG$_6$**	7.18	95	20	1136.2 (1136.5)
**[C^{10}(chol)]PN9scram**	6.20[b]	95	18	869.2 (869.2)
**[C^{15}(chol)]PN9scramble-spacer-PEG$_6$**	7.15	90	20	1210.0 (1209.7)
**[C^{15}(chol)]PN9-spacer-PEG$_{12}$**	7.45	95	19	895.3 (895.1)[e]

Chromatographic data

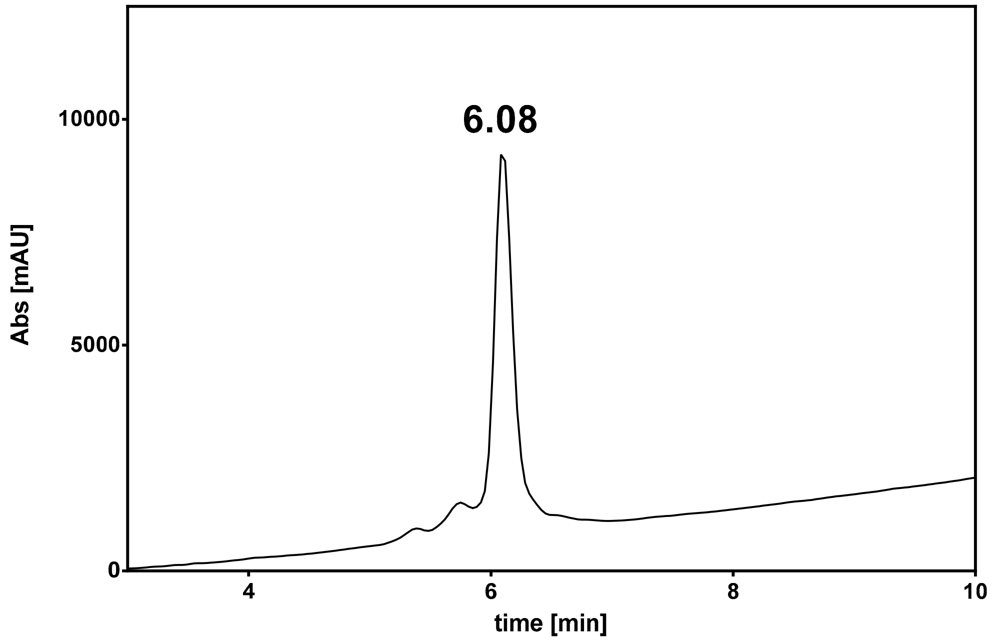

Figure S1. RP-HPLC traces of the peptide [C[1](chol)]PN19. Conditions: C4 column Kromasil (5 µm × 4.6 × 150 mm); temperature, 308 K; flow, 1 mL/min; eluents, 0.1% (*v*/*v*) TFA in H_2O (A) and 0.1% (*v*/*v*) TFA in ACN (B); λ, 215 nm; gradient, 70 - 100% B in A over 10 min. R_t = 6.08 min.

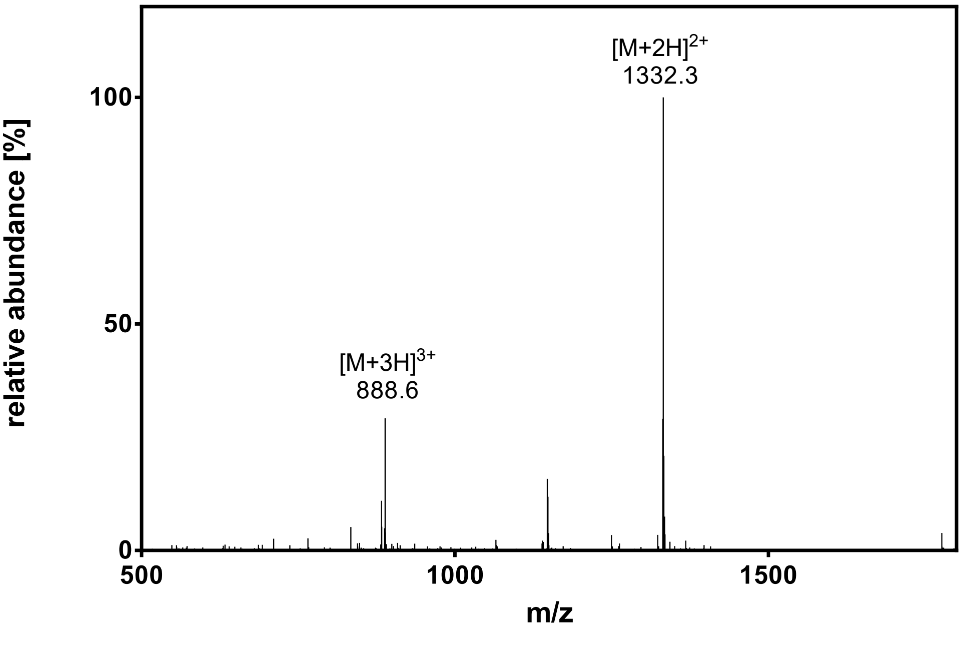

Figure S2. ESI-MS spectrum [C[1](chol)]PN19. ESI-MS (m/z): $[M + 2H]^{2+}$ 1332.3 (found), 1332.2 (calcd).

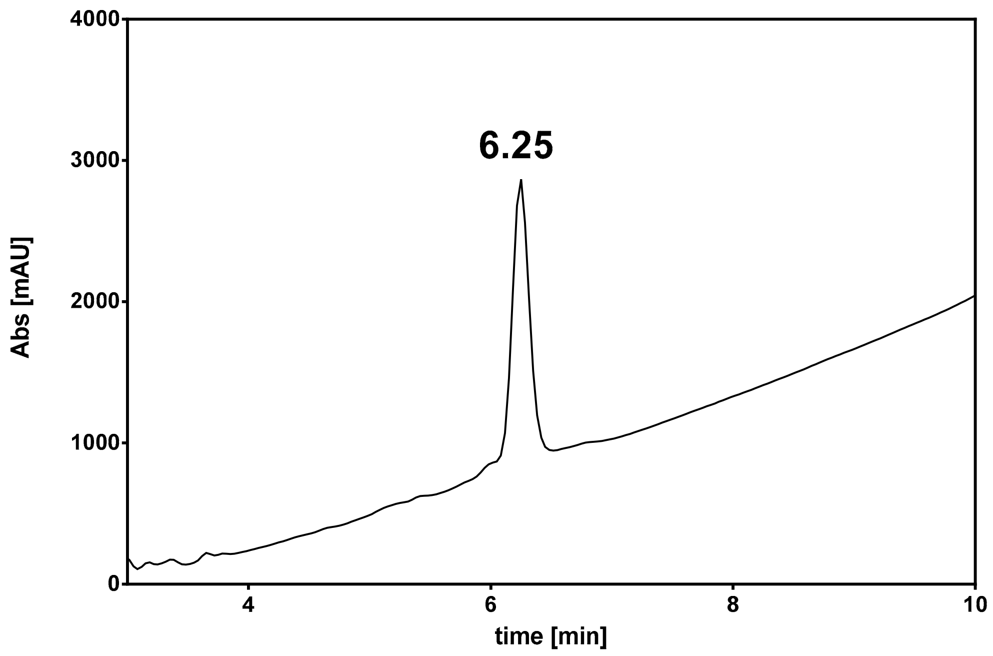

Figure S3. RP-HPLC traces of the peptide [C^{20}(chol)]PN19. Conditions: C4 column Kromasil (5 μm × 4.6 × 150 mm); temperature, 308 K; flow, 1 mL/min; eluents, 0.1% (*v*/*v*) TFA in H_2O (A) and 0.1% (*v*/*v*) TFA in ACN (B); λ, 215 nm; gradient, 70 - 100% B in A over 10 min. R_t = 6.25 min.

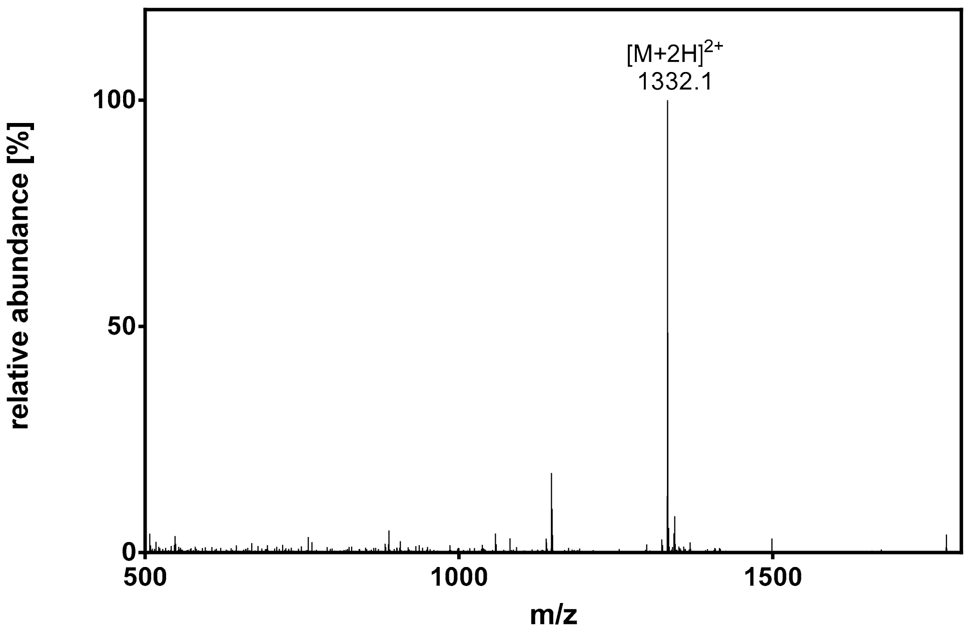

Figure S4. ESI-MS spectrum [C^{20}(chol)]PN19. ESI-MS (m/z): $[M + 2H]^{2+}$ 1332.1 (found), 1332.2 (calcd).

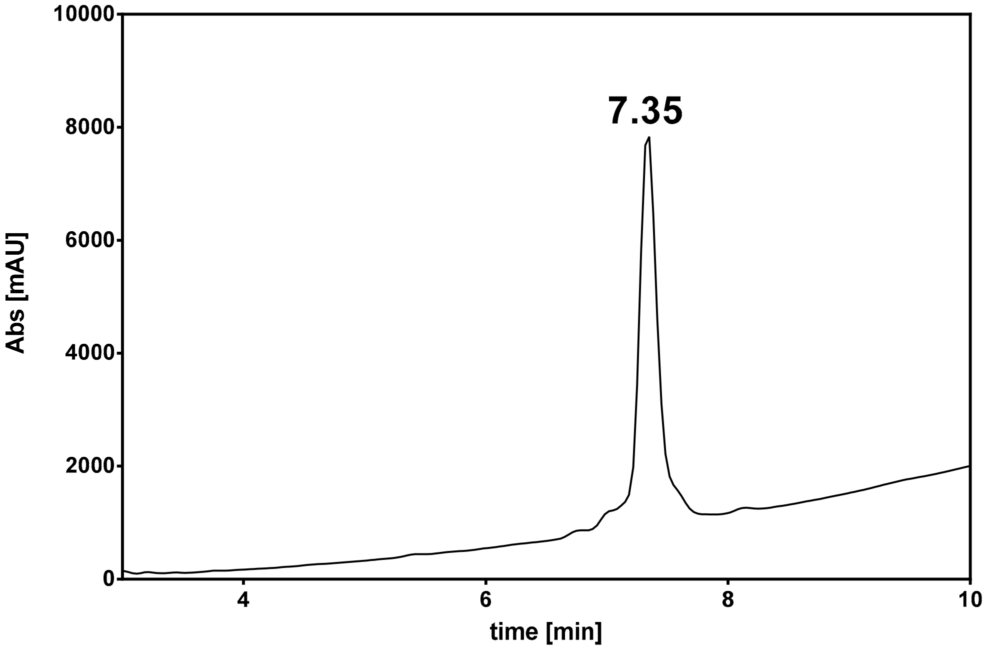

Figure S5. RP-HPLC traces of the peptide [C[1](chol)]PN19-spacer. Conditions: C4 column Kromasil (5 µm × 4.6 × 150 mm); temperature, 308 K; flow, 1 mL/min; eluents, 0.1% (*v*/*v*) TFA in H_2O (A) and 0.1% (*v*/*v*) TFA in ACN (B); λ, 215 nm; gradient, 50 - 100% B in A over 10 min. R_t = 7.35 min.

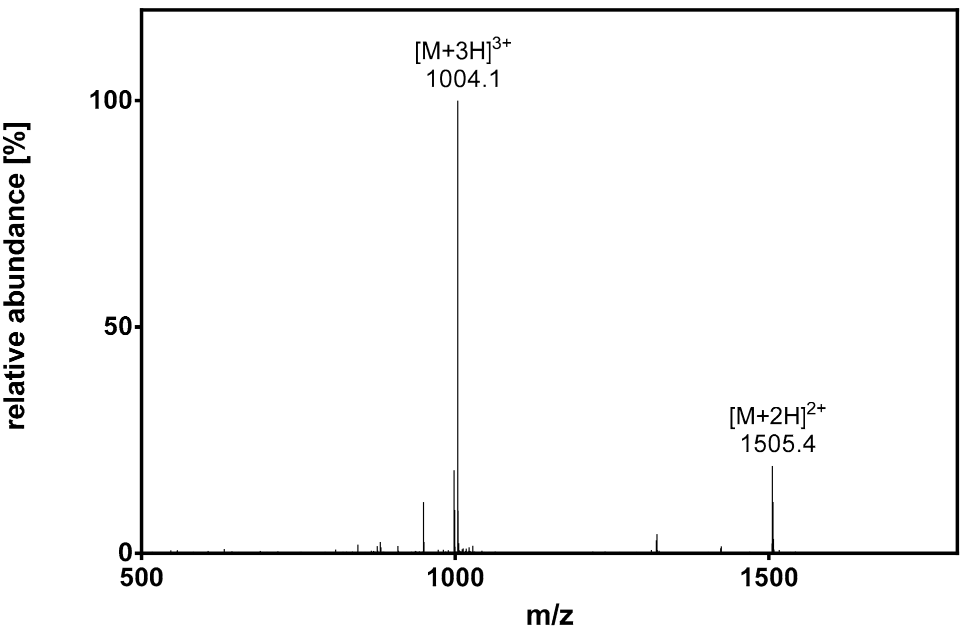

Figure S6. ESI-MS spectrum [C[1](chol)]PN19-spacer. ESI-MS (m/z): $[M + 3H]^{3+}$ 1004.1 (found), 1004.3 (calcd).

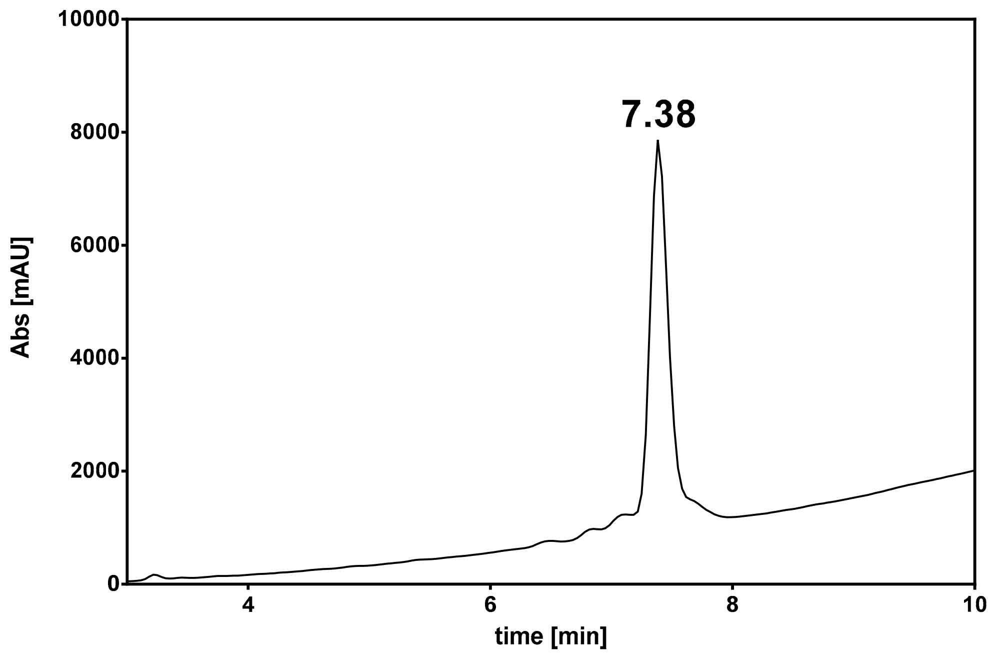

Figure S7. RP-HPLC traces of the peptide [C^{25}(chol)]PN19-spacer. Conditions: C4 column Kromasil (5 µm × 4.6 × 150 mm); temperature, 308 K; flow, 1 mL/min; eluents, 0.1% (*v*/*v*) TFA in H_2O (A) and 0.1% (*v*/*v*) TFA in ACN (B); λ, 215 nm; gradient, 50 - 100% B in A over 10 min. R_t = 7.38 min.

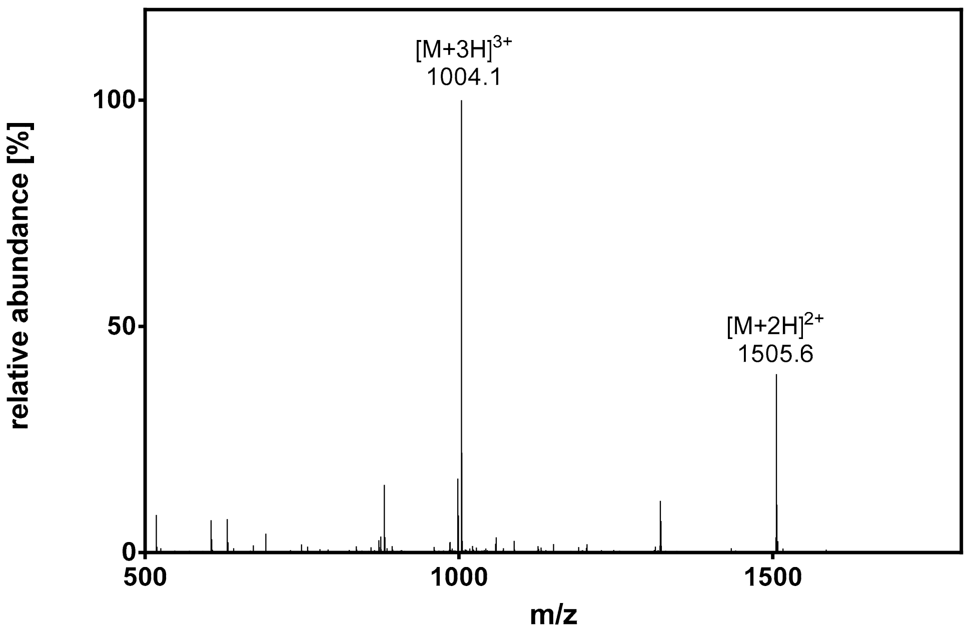

Figure S8. ESI-MS spectrum [C^{25}(chol)]PN19-spacer. ESI-MS (m/z): $[M + 3H]^{3+}$ 1004.1 (found), 1004.3 (calcd).

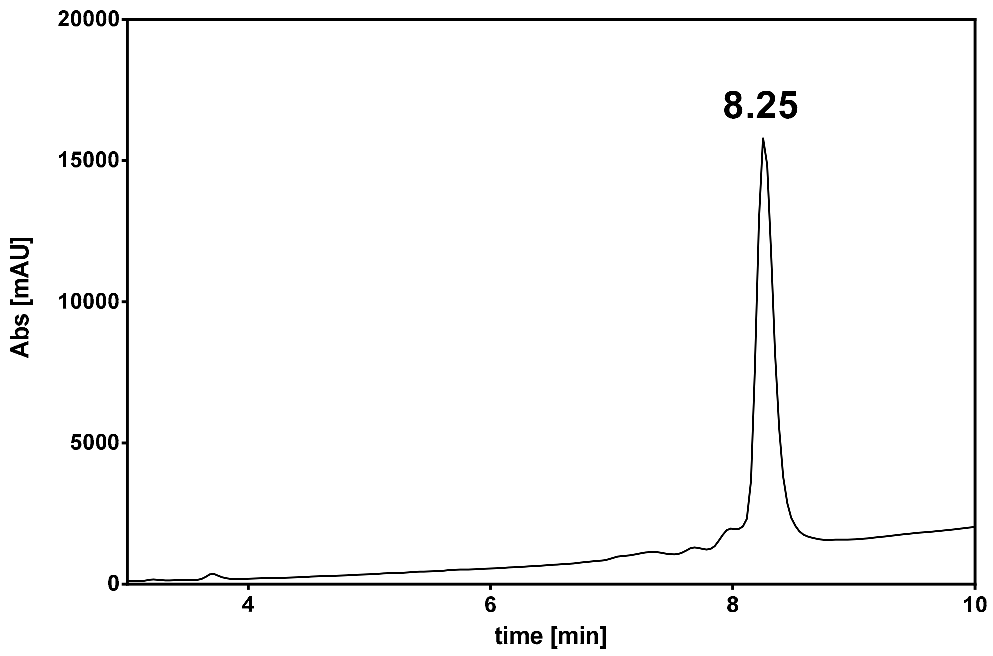

Figure S9. RP-HPLC traces of the peptide [C[1](chol)]PN19-PEG6. Conditions: C4 column Kromasil (5 µm × 4.6 × 150 mm); temperature, 308 K; flow, 1 mL/min; eluents, 0.1% (*v*/*v*) TFA in H_2O (A) and 0.1% (*v*/*v*) TFA in ACN (B); λ, 215 nm; gradient, 50 - 100% B in A over 10 min. R_t = 8.25 min.

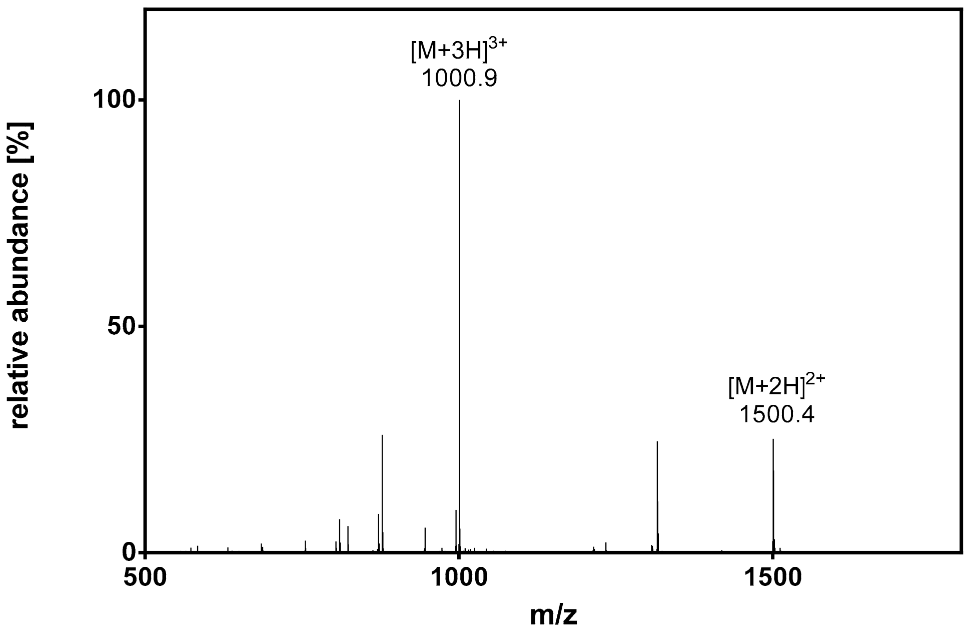

Figure S10. ESI-MS spectrum [C[1](chol)]PN19-PEG6. ESI-MS (m/z): $[M + 3H]^{3+}$ 1000.9 (found), 1000.7 (calcd).

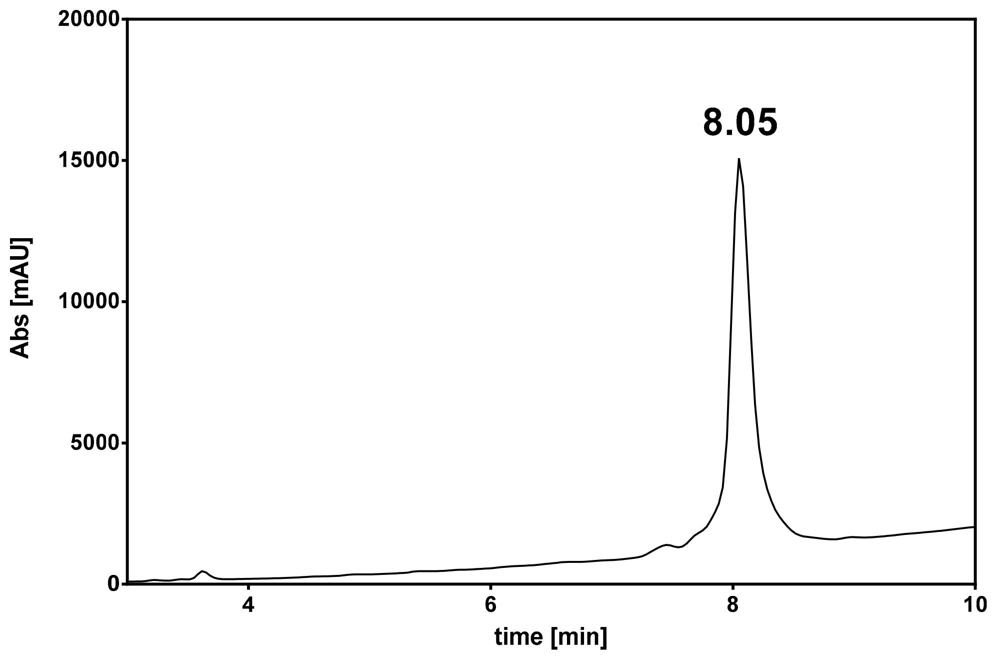

Figure S11. RP-HPLC traces of the peptide [C^{20}(chol)]PN19-PEG_6. Conditions: C4 column Kromasil (5 µm × 4.6 × 150 mm); temperature, 308 K; flow, 1 mL/min; eluents, 0.1% (*v*/*v*) TFA in H_2O (A) and 0.1% (*v*/*v*) TFA in ACN (B); λ, 215 nm; gradient, 50 - 100% B in A over 10 min. R_t = 8.05 min.

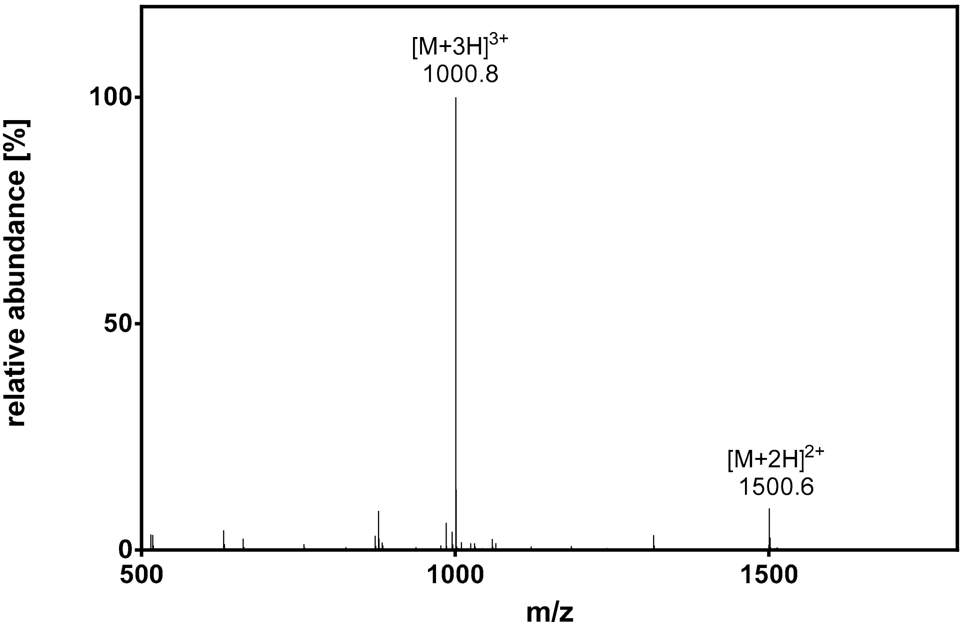

Figure S12. ESI-MS spectrum [C^{20}(chol)]PN19-PEG_6. ESI-MS (m/z): $[M + 3H]^{3+}$ 1000.8 (found), 1000.7 (calcd).

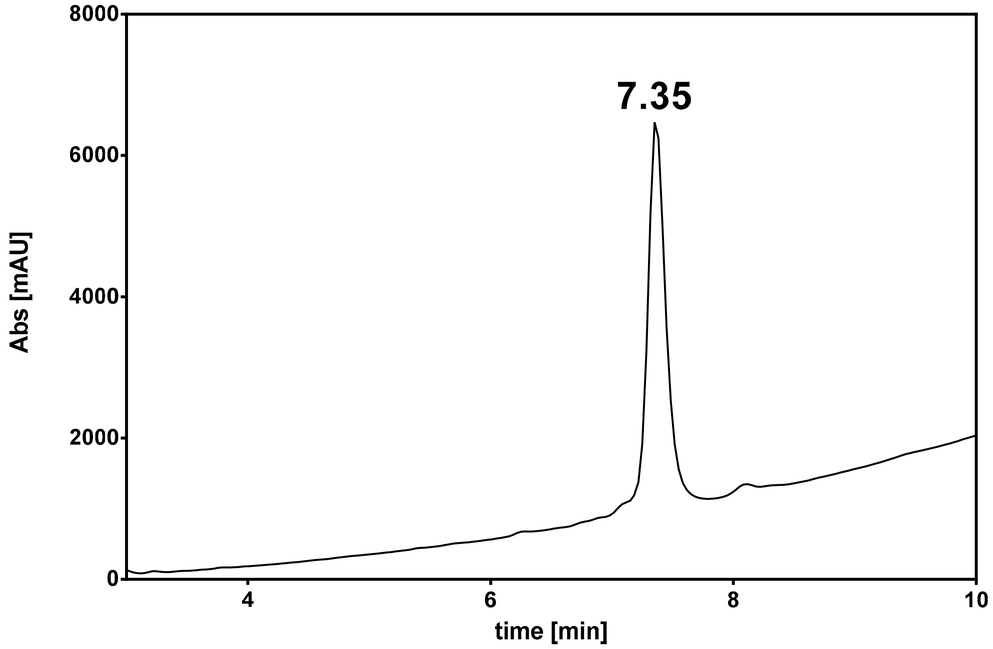

Figure S13. RP-HPLC traces of the peptide [C[1](chol)]PN19-spacer-PEG6. Conditions: C4 column Kromasil (5 μm × 4.6 × 150 mm); temperature, 308 K; flow, 1 mL/min; eluents, 0.1% (*v*/*v*) TFA in H_2O (A) and 0.1% (*v*/*v*) TFA in ACN (B); λ, 215 nm; gradient, 50 - 100% B in A over 10 min. R_t = 7.35 min.

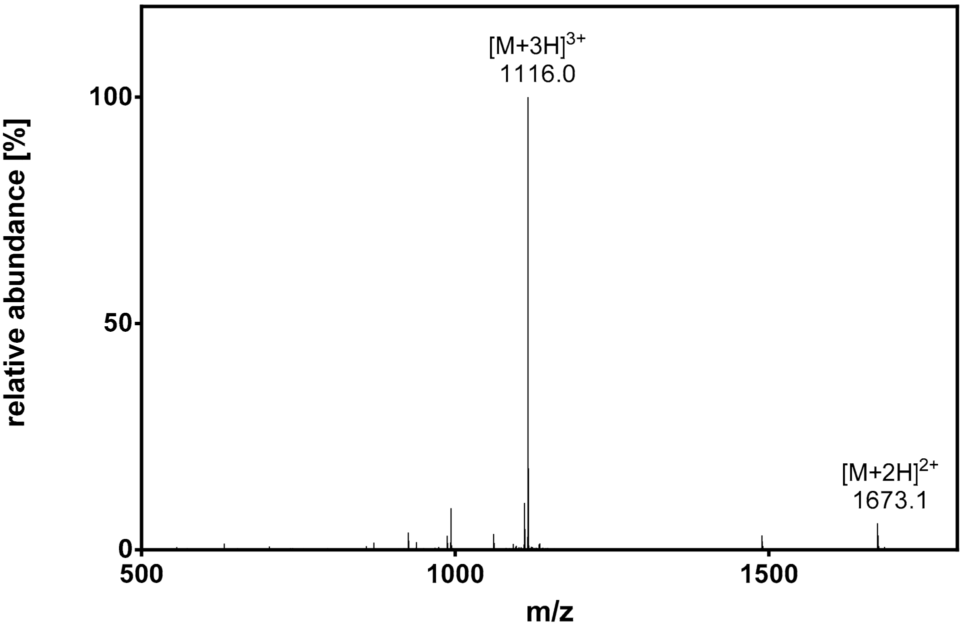

Figure S14. ESI-MS spectrum [C[1](chol)]PN19-spacer-PEG6. ESI-MS (m/z): $[M + 3H]^{3+}$ 1116.0 (found), 1116.2 (calcd).

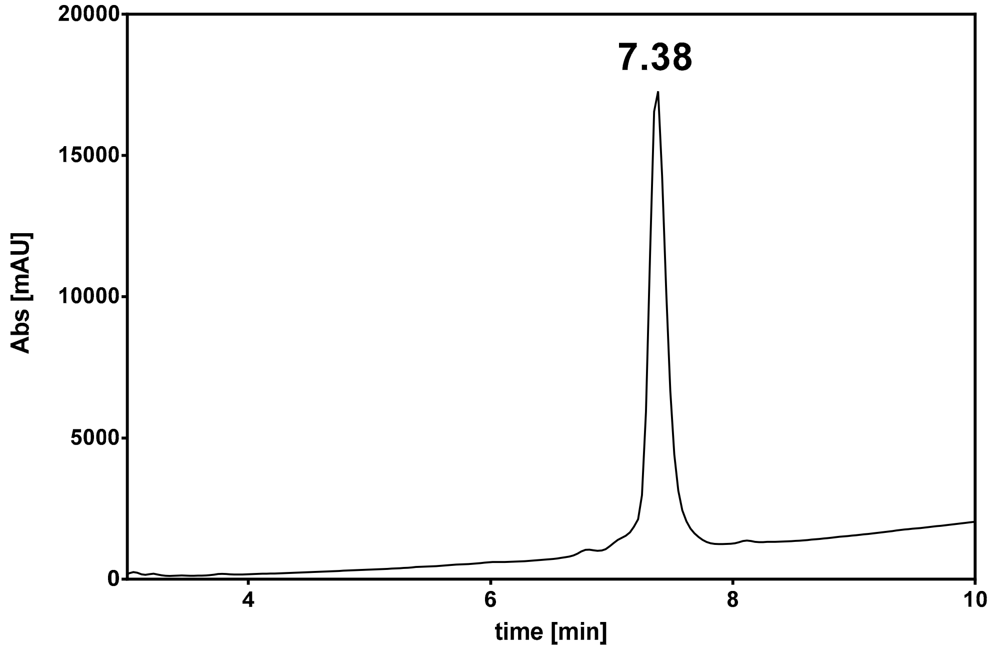

Figure S15. RP-HPLC traces of the peptide [C^{25}(chol)]PN19-spacer-PEG_6. Conditions: C4 column Kromasil (5 µm × 4.6 × 150 mm); temperature, 308 K; flow, 1 mL/min; eluents, 0.1% (*v*/*v*) TFA in H_2O (A) and 0.1% (*v*/*v*) TFA in ACN (B); λ, 215 nm; gradient, 50 - 100% B in A over 10 min. R_t = 7.38 min.

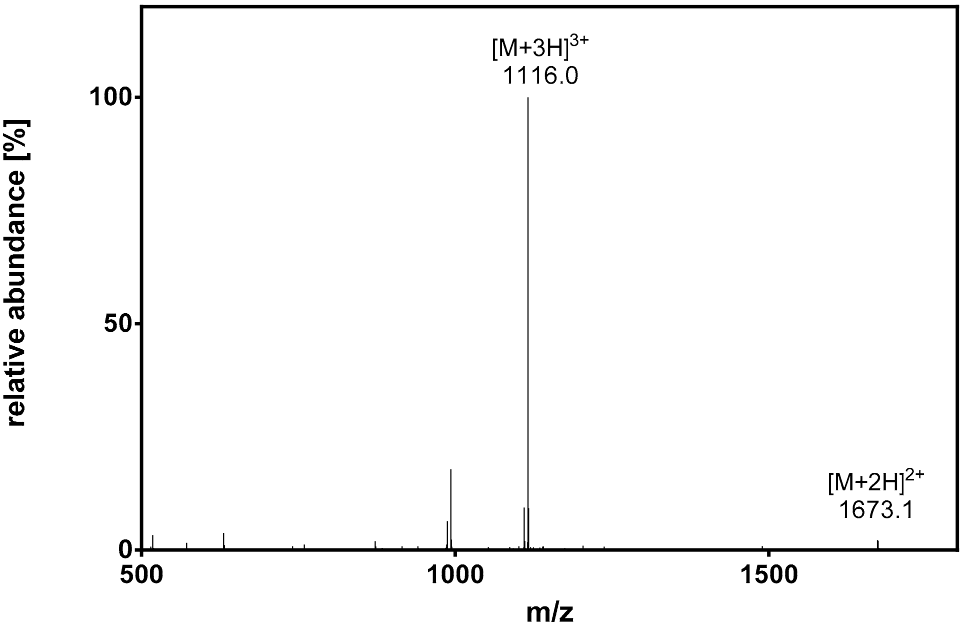

Figure S16. ESI-MS spectrum [C^{25}(chol)]PN19-spacer-PEG_6. ESI-MS (m/z): $[M + 3H]^{3+}$ 1116.0 (found), 1116.2 (calcd).

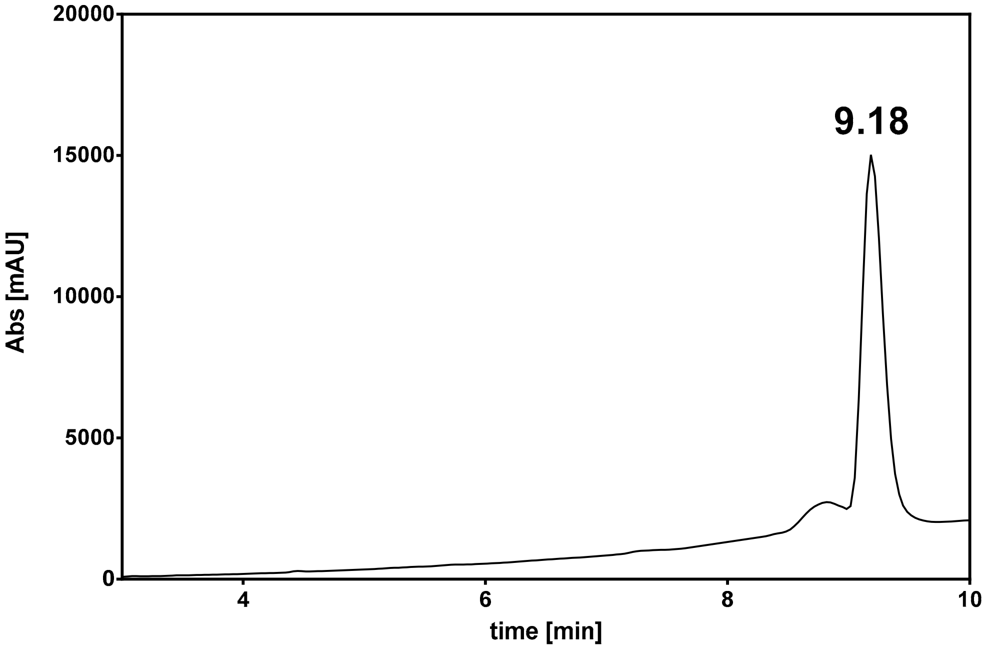

Figure S17. RP-HPLC traces of the peptide [C[1](chol)]PN13. Conditions: C4 column Kromasil (5 μm × 4.6 × 150 mm); temperature, 308 K; flow, 1 mL/min; eluents, 0.1% (*v*/*v*) TFA in H_2O (A) and 0.1% (*v*/*v*) TFA in ACN (B); λ, 215 nm; gradient, 50 - 100% B in A over 10 min. R_t = 9.18 min.

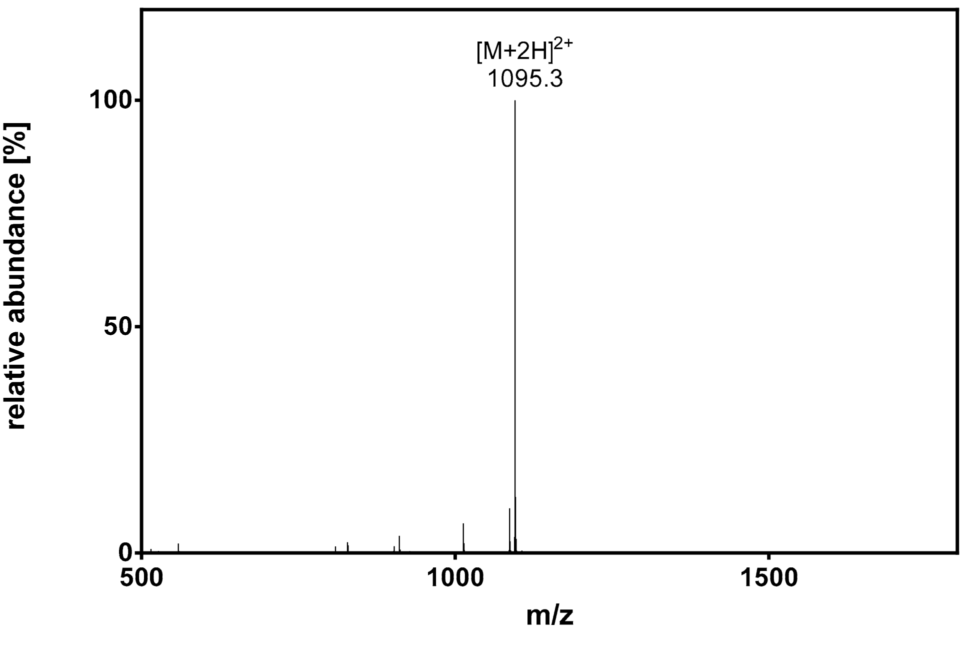

Figure S18. ESI-MS spectrum [C[1](chol)]PN13. ESI-MS (m/z): $[M + 2H]^{2+}$ 1095.3 (found), 1095.2 (calcd).

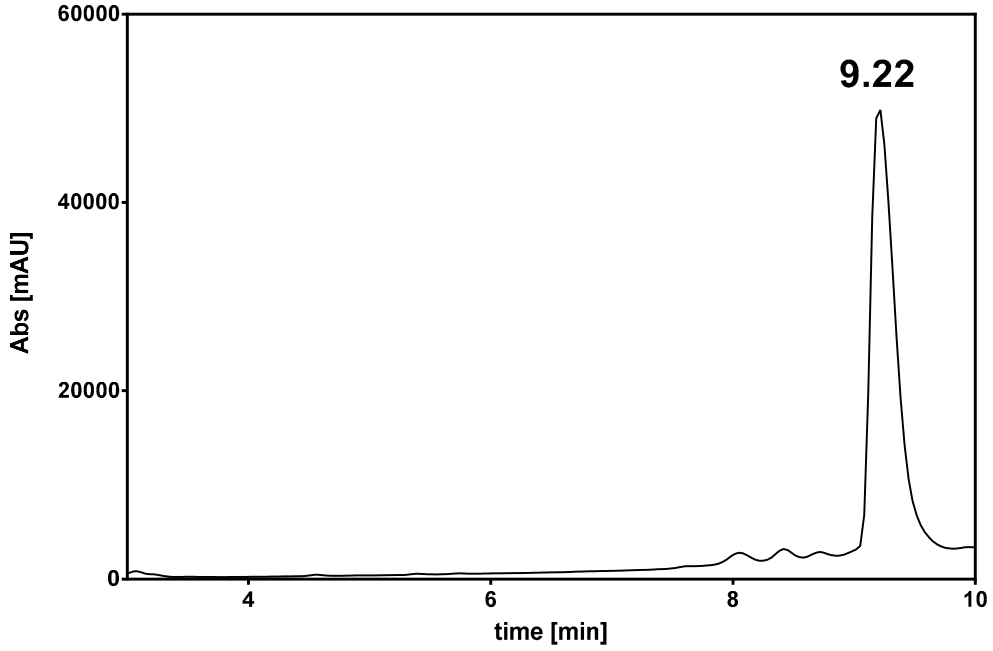

Figure S19. RP-HPLC traces of the peptide [C^{14}(chol)]PN13. Conditions: C4 column Kromasil (5 μm × 4.6 × 150 mm); temperature, 308 K; flow, 1 mL/min; eluents, 0.1% (*v*/*v*) TFA in H_2O (A) and 0.1% (*v*/*v*) TFA in ACN (B); λ, 215 nm; gradient, 50 - 100% B in A over 10 min. R_t = 9.22 min.

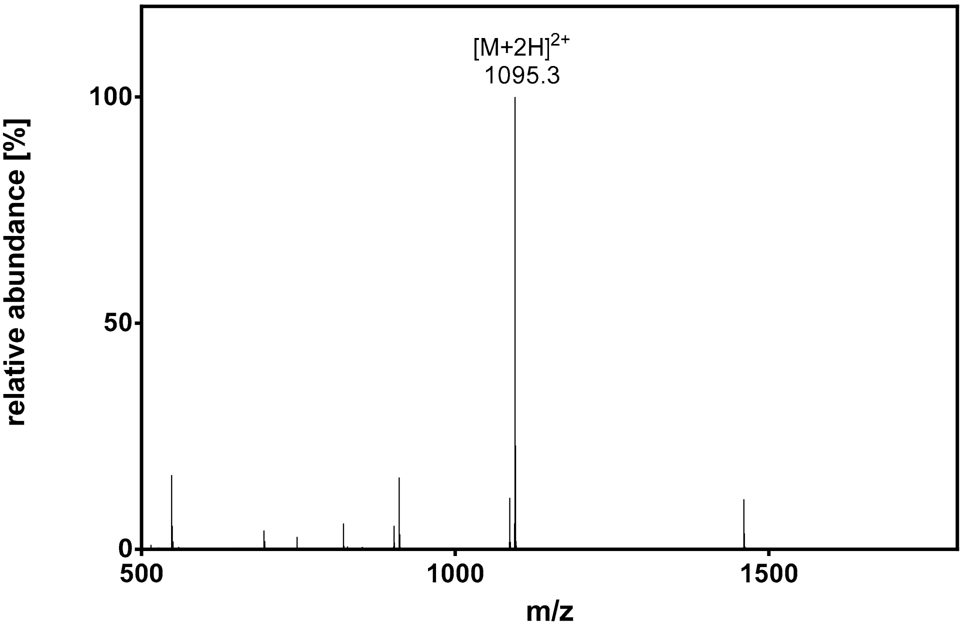

Figure S20. ESI-MS spectrum [C^{14}(chol)]PN13. ESI-MS (m/z): $[M + 2H]^{2+}$ 1095.3 (found), 1095.2 (calcd).

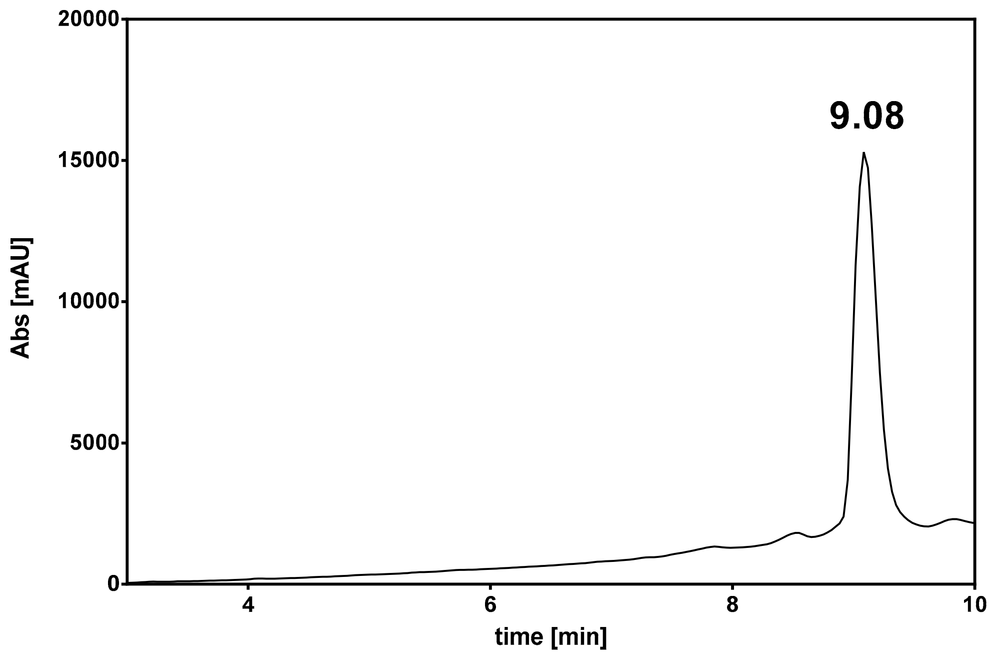

Figure S21. RP-HPLC traces of the peptide [C[1](chol)]PN9. Conditions: C4 column Kromasil (5 μm × 4.6 × 150 mm); temperature, 308 K; flow, 1 mL/min; eluents, 0.1% (*v*/*v*) TFA in H_2O (A) and 0.1% (*v*/*v*) TFA in ACN (B); λ, 215 nm; gradient, 50 - 100% B in A over 10 min. R_t = 9.08 min.

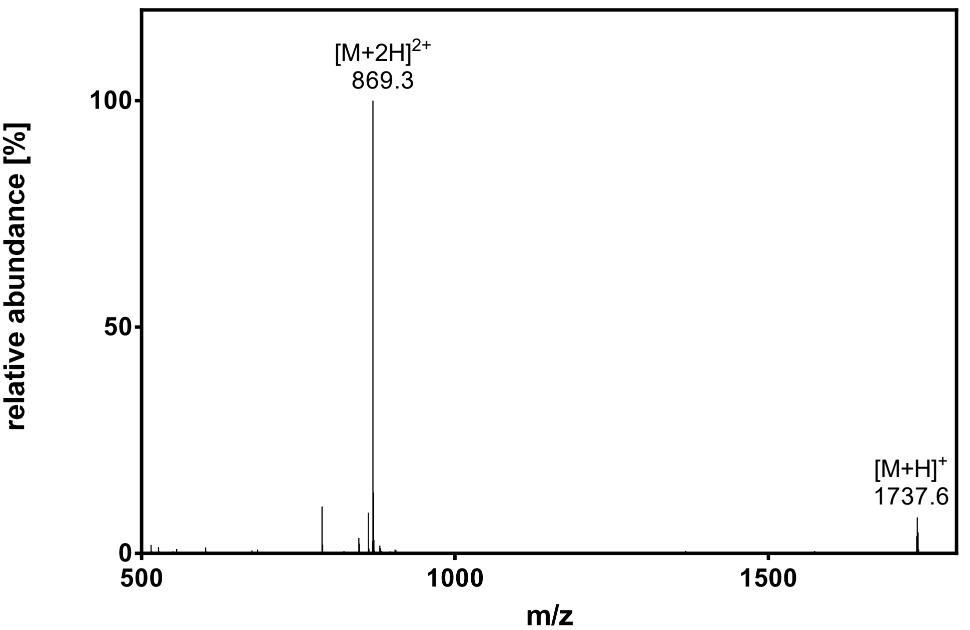

Figure S22. ESI-MS spectrum [C[1](chol)]PN9. ESI-MS (m/z): $[M + 2H]^{2+}$ 869.3 (found), 869.2 (calcd).

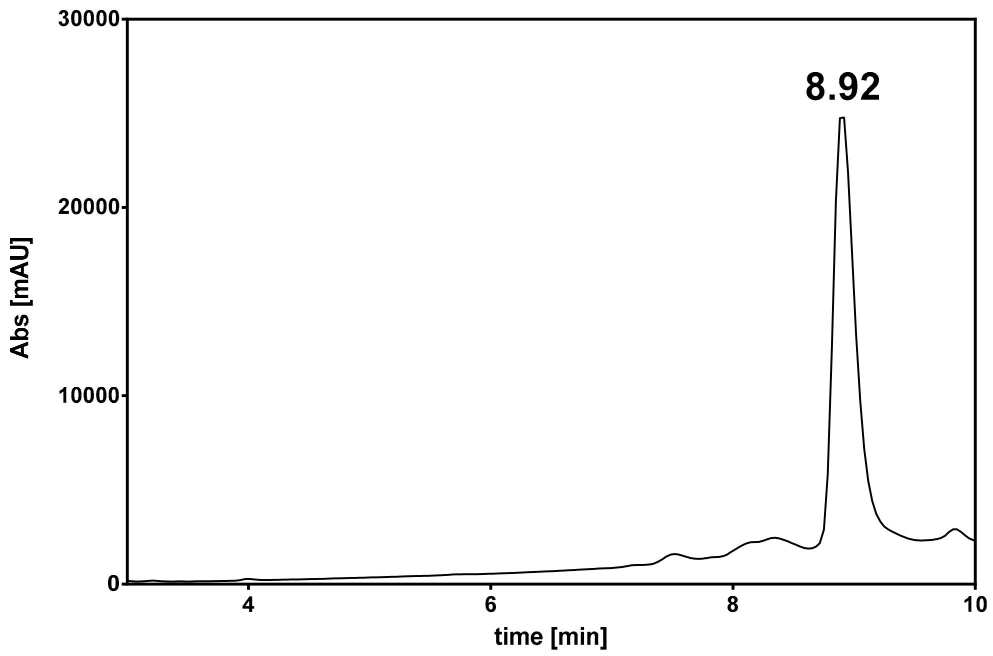

Figure S23. RP-HPLC traces of the peptide [C^{10}(chol)]PN9. Conditions: C4 column Kromasil (5 µm × 4.6 × 150 mm); temperature, 308 K; flow, 1 mL/min; eluents, 0.1% (*v*/*v*) TFA in H_2O (A) and 0.1% (*v*/*v*) TFA in ACN (B); λ, 215 nm; gradient, 50 - 100% B in A over 10 min. R_t = 8.92 min.

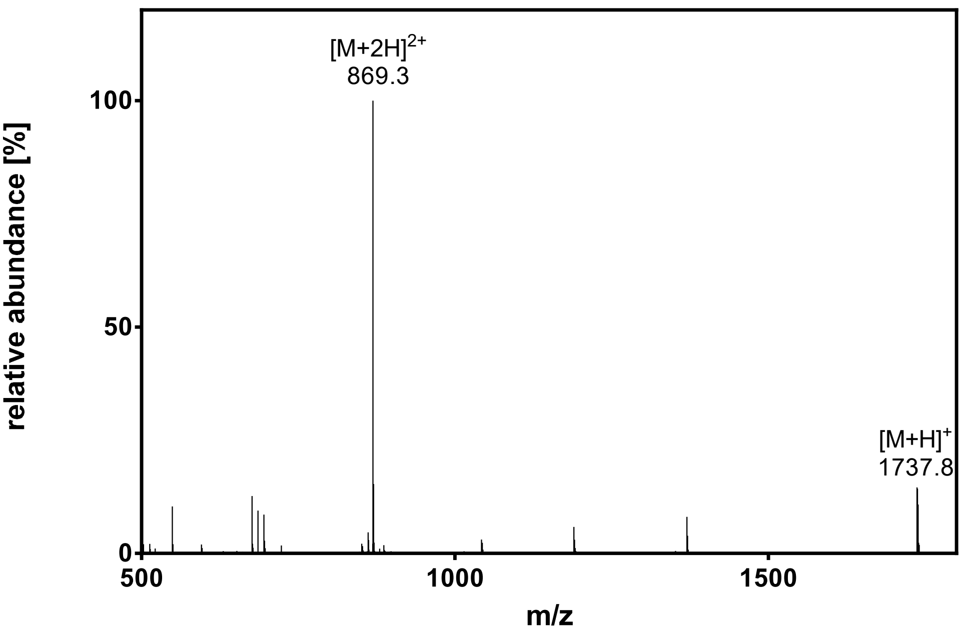

Figure S24. ESI-MS spectrum [C^{10}(chol)]PN9. ESI-MS (m/z): $[M + 2H]^{2+}$ 869.3 (found), 869.2 (calcd).

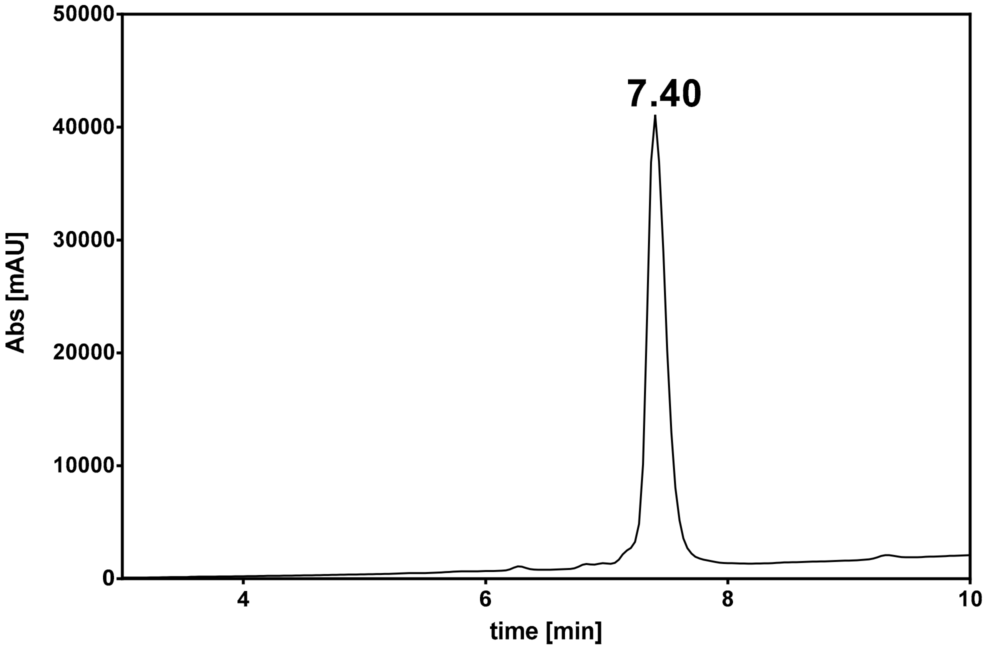

Figure S25. RP-HPLC traces of the peptide [C[1](chol)]PN13-spacer-PEG$_6$. Conditions: C4 column Kromasil (5 µm × 4.6 × 150 mm); temperature, 308 K; flow, 1 mL/min; eluents, 0.1% (*v*/*v*) TFA in H_2O (A) and 0.1% (*v*/*v*) TFA in ACN (B); λ, 215 nm; gradient, 50 - 100% B in A over 10 min. R_t = 7.40 min.

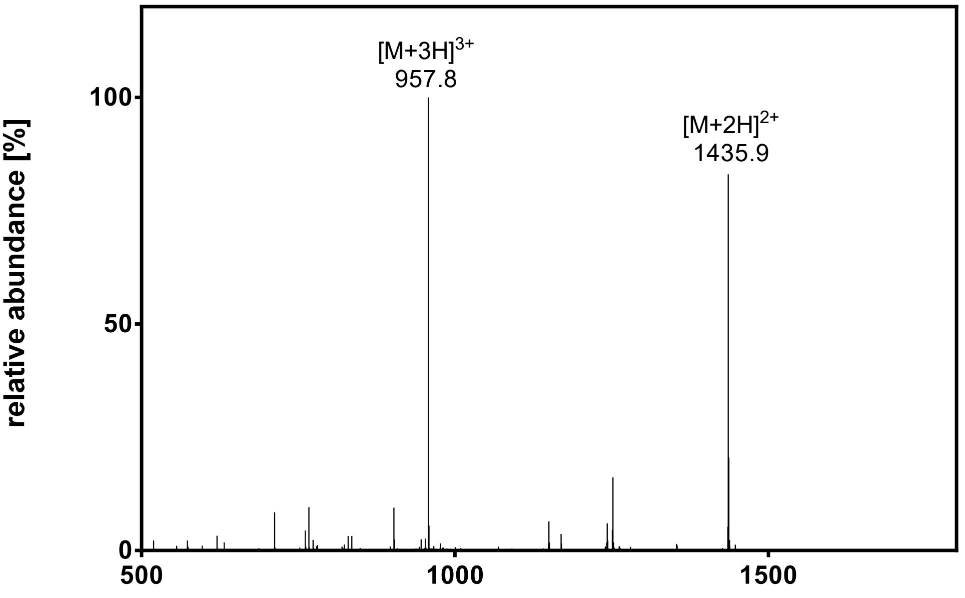

Figure S26. ESI-MS spectrum [C[1](chol)]PN13-spacer-PEG$_6$. ESI-MS (m/z): $[M + 3H]^{3+}$ 957.8 (found), 957.6 (calcd).

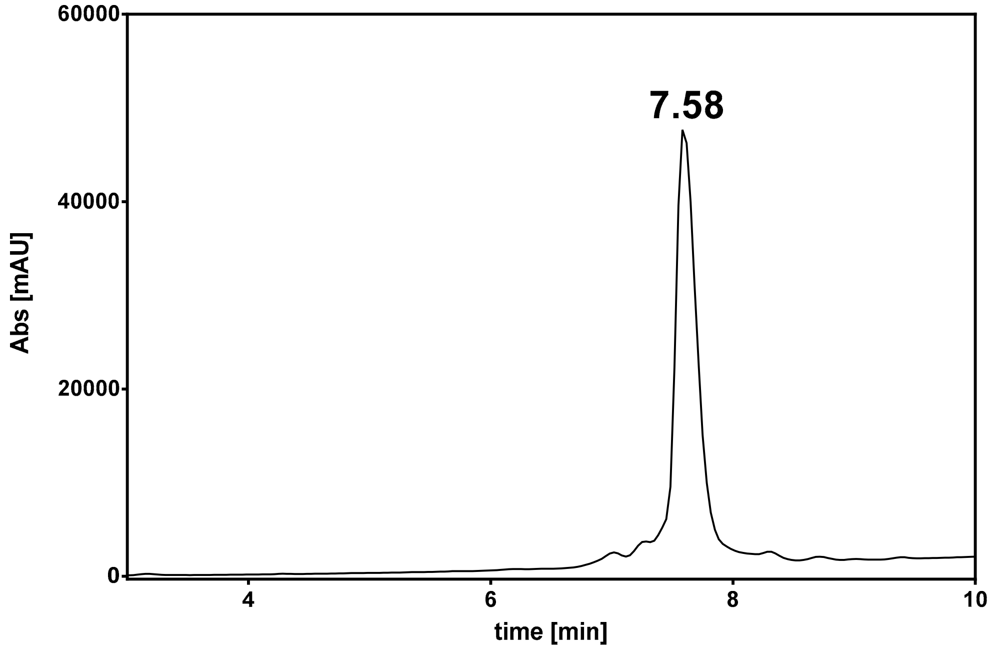

Figure S27. RP-HPLC traces of the peptide [C^{19}(chol)]PN13-spacer-PEG_6. Conditions: C4 column Kromasil (5 µm × 4.6 × 150 mm); temperature, 308 K; flow, 1 mL/min; eluents, 0.1% (*v*/*v*) TFA in H_2O (A) and 0.1% (*v*/*v*) TFA in ACN (B); λ, 215 nm; gradient, 50 - 100% B in A over 10 min. R_t = 7.58 min.

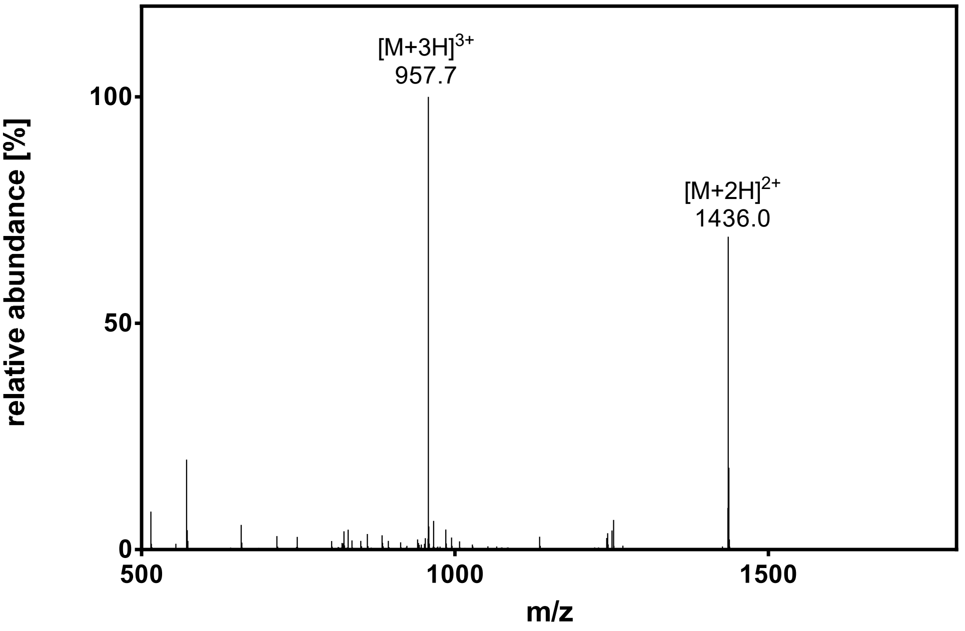

Figure S28. ESI-MS spectrum [C^{19}(chol)]PN13-spacer-PEG_6. ESI-MS (m/z): $[M + 3H]^{3+}$ 957.7 (found), 957.6 (calcd).

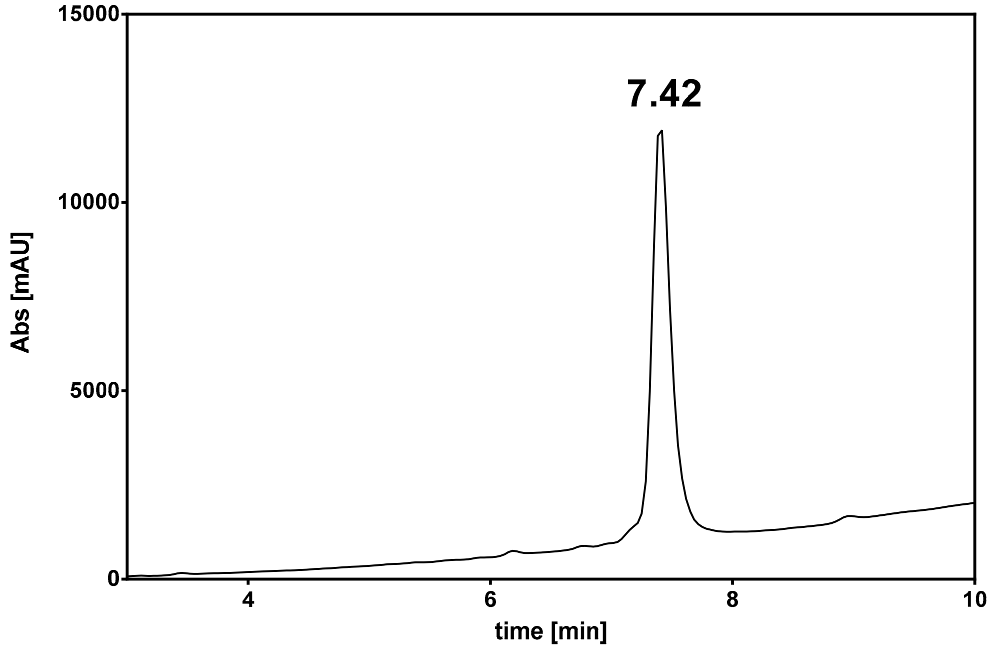

Figure S29. RP-HPLC traces of the peptide [C[1](chol)]PN9-spacer-PEG6. Conditions: C4 column Kromasil (5 µm × 4.6 × 150 mm); temperature, 308 K; flow, 1 mL/min; eluents, 0.1% (*v*/*v*) TFA in H_2O (A) and 0.1% (*v*/*v*) TFA in ACN (B); λ, 215 nm; gradient, 50 - 100% B in A over 10 min. R_t = 7.42 min.

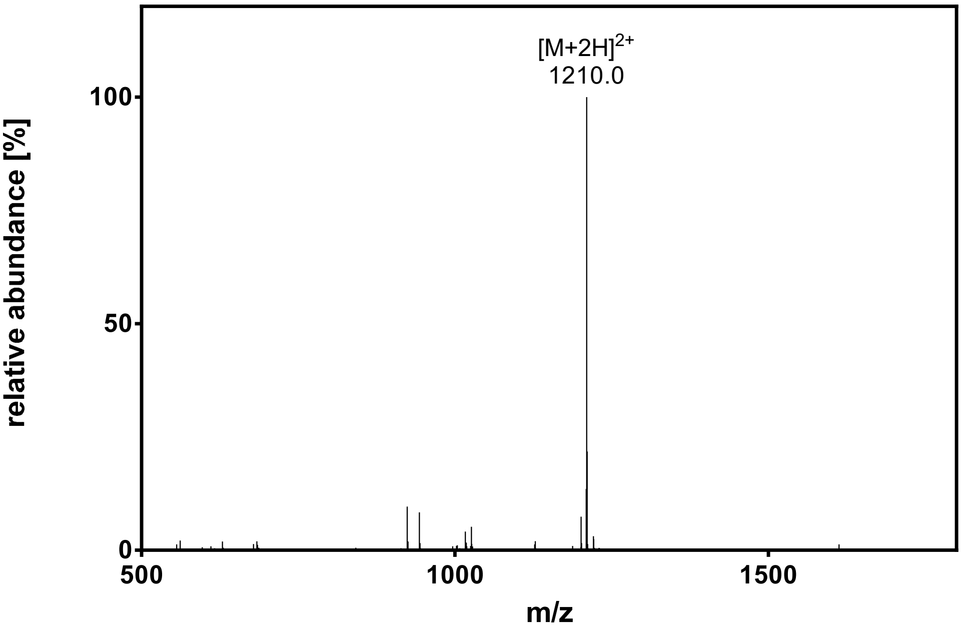

Figure S30. ESI-MS spectrum [C[1](chol)]PN9-spacer-PEG6. ESI-MS (m/z): $[M + 2H]^{2+}$ 1210.0 (found), 1209.7 (calcd).

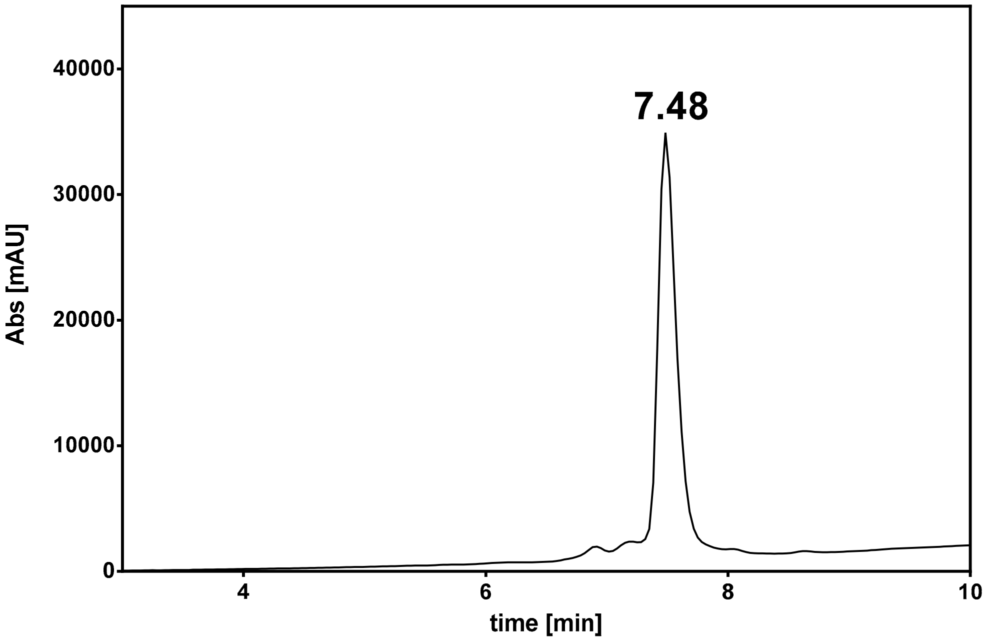

Figure S31. RP-HPLC traces of the peptide [C[15](chol)]PN9-spacer-PEG$_6$. Conditions: C4 column Kromasil (5 µm × 4.6 × 150 mm); temperature, 308 K; flow, 1 mL/min; eluents, 0.1% (*v*/*v*) TFA in H_2O (A) and 0.1% (*v*/*v*) TFA in ACN (B); λ, 215 nm; gradient, 50 - 100% B in A over 10 min. R_t = 7.48 min.

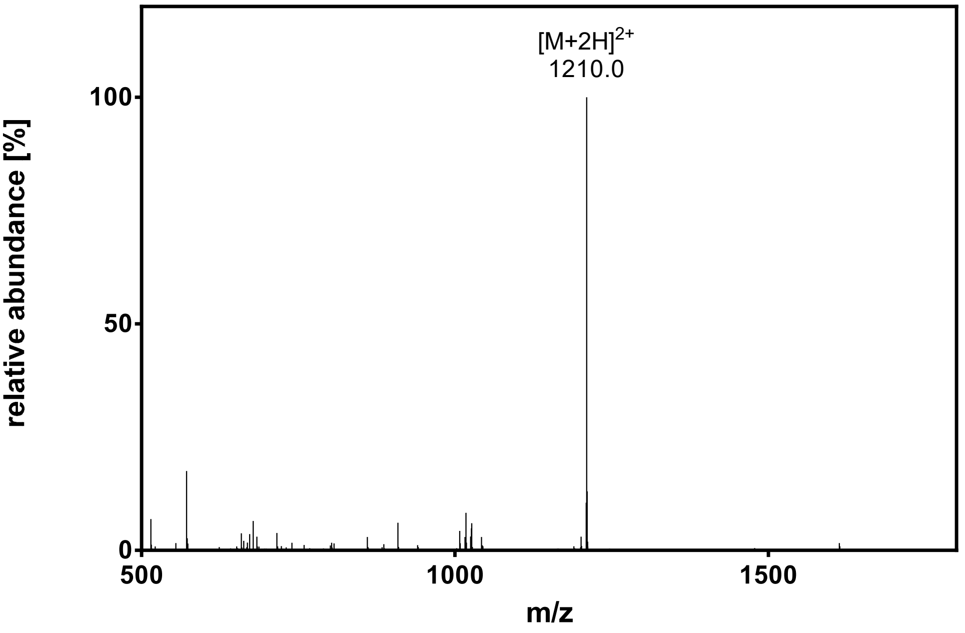

Figure S32. ESI-MS spectrum [C[15](chol)]PN9-spacer-PEG$_6$. ESI-MS (m/z): $[M + 2H]^{2+}$ 1210.0 (found), 1209.7 (calcd).

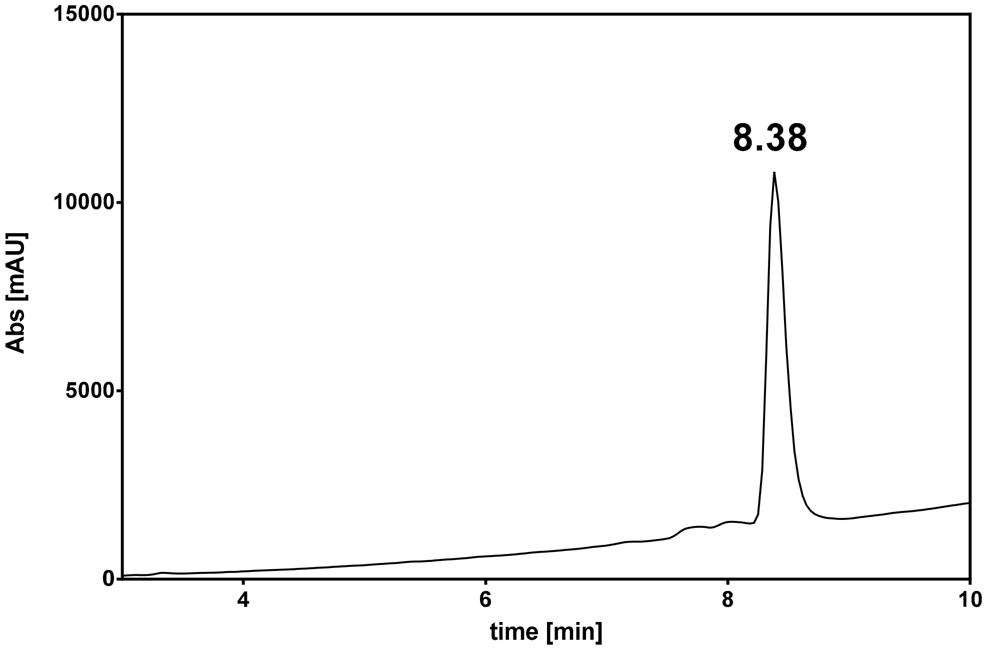

Figure S33. RP-HPLC traces of the peptide [C[9](chol)]PN8. Conditions: C4 column Kromasil (5 µm × 4.6 × 150 mm); temperature, 308 K; flow, 1 mL/min; eluents, 0.1% (*v*/*v*) TFA in H_2O (A) and 0.1% (*v*/*v*) TFA in ACN (B); λ, 215 nm; gradient, 50 - 100% B in A over 10 min. R_t = 8.38 min.

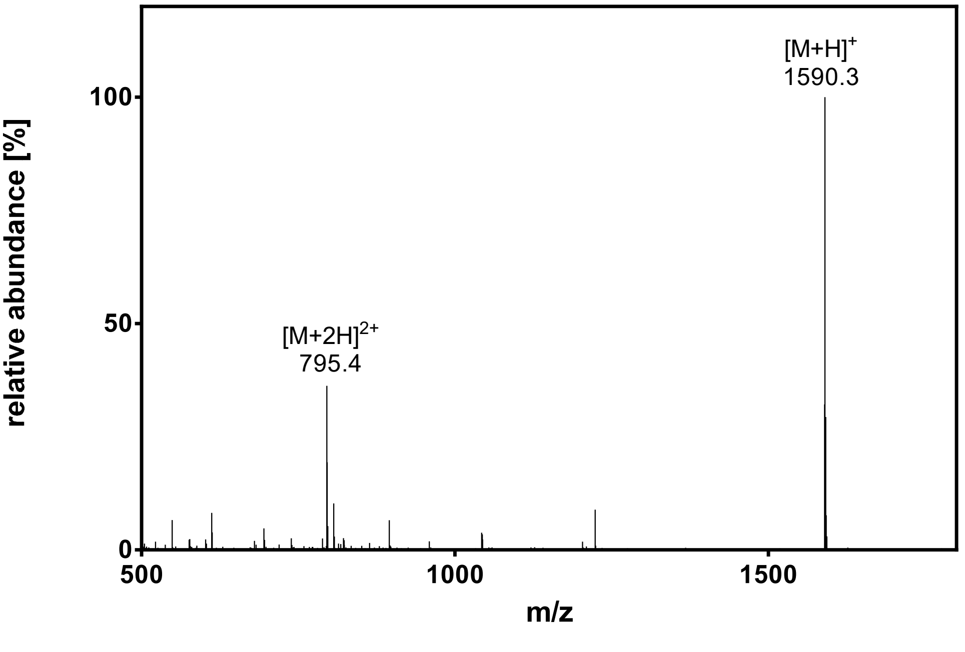

Figure S34. ESI-MS spectrum [C[9](chol)]PN8. ESI-MS (m/z): $[M + H]^+$ 1590.3 (found), 1590.1 (calcd).

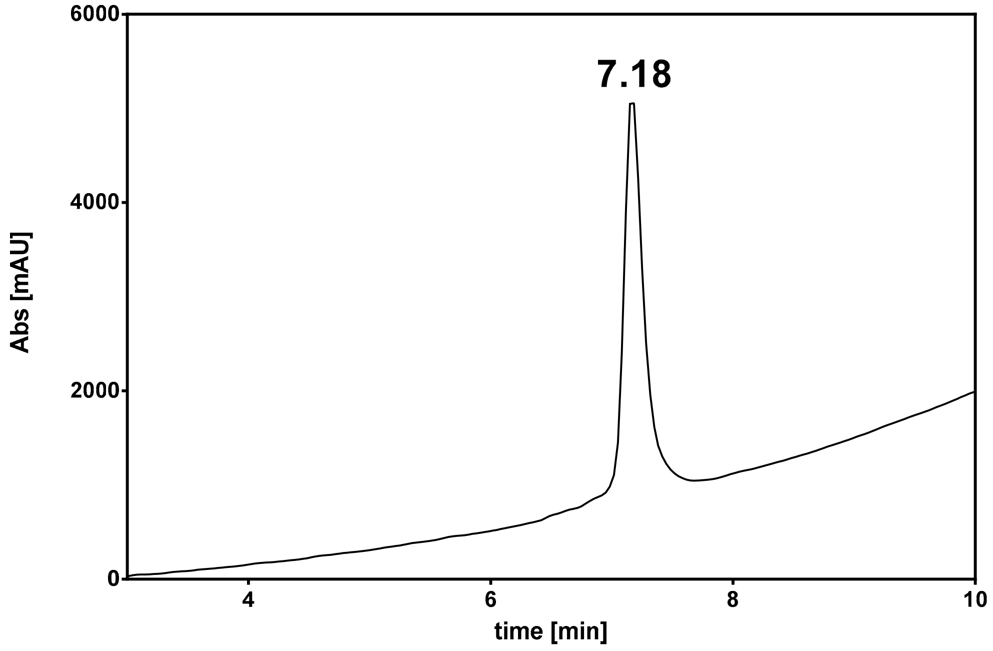

Figure S35. RP-HPLC traces of the peptide [C^{14}(chol)]PN8-spacer-PEG_6. Conditions: C4 column Kromasil (5 μm × 4.6 × 150 mm); temperature, 308 K; flow, 1 mL/min; eluents, 0.1% (*v*/*v*) TFA in H_2O (A) and 0.1% (*v*/*v*) TFA in ACN (B); λ, 215 nm; gradient, 50 - 100% B in A over 10 min. R_t = 7.18 min.

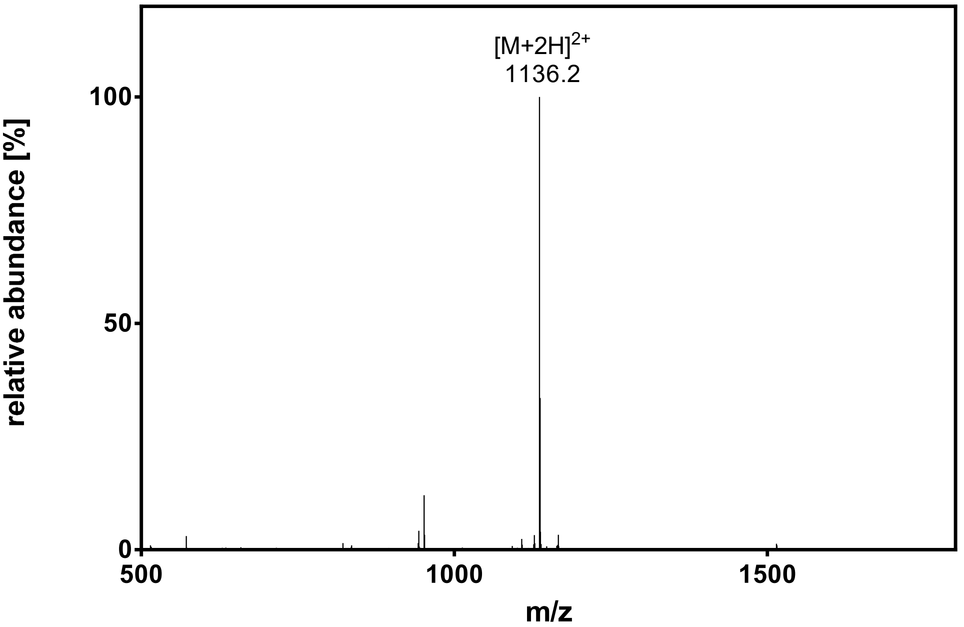

Figure S36. ESI-MS spectrum [C^{14}(chol)]PN8-spacer-PEG_6. ESI-MS (m/z): $[M + 2H]^{2+}$ 1136.2 (found), 1136.5 (calcd).

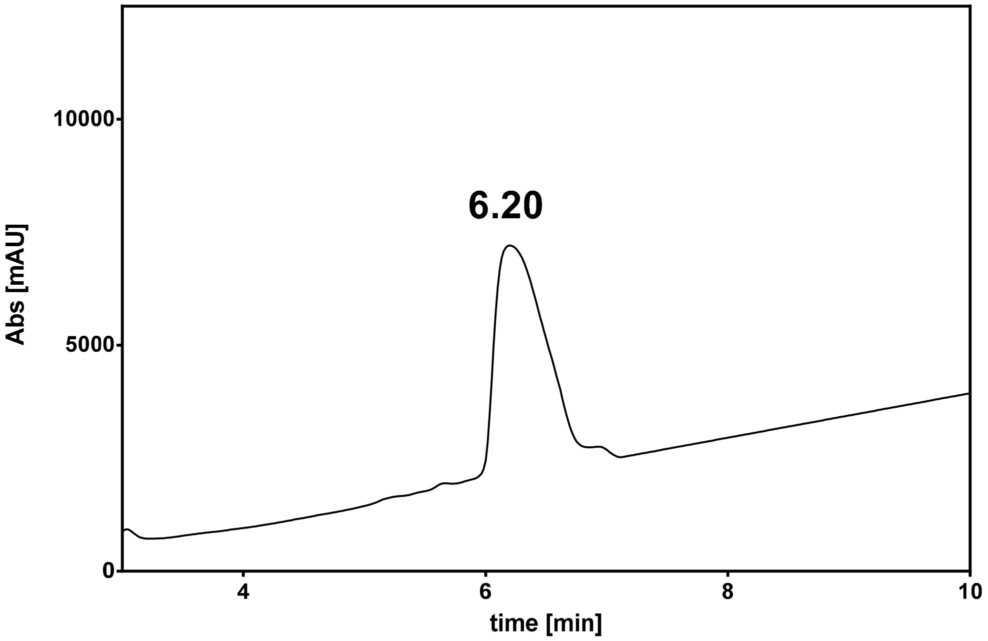

Figure S37. RP-HPLC traces of the peptide [C^{10}(chol)]PN9scram. Conditions: C4 column Kromasil (5 µm × 4.6 × 150 mm); temperature, 308 K; flow, 1 mL/min; eluents, 0.1% (*v*/*v*) TFA in H_2O (A) and 0.1% (*v*/*v*) TFA in ACN (B); λ, 215 nm; gradient, 70 - 100% B in A over 10 min. R_t = 6.20 min.

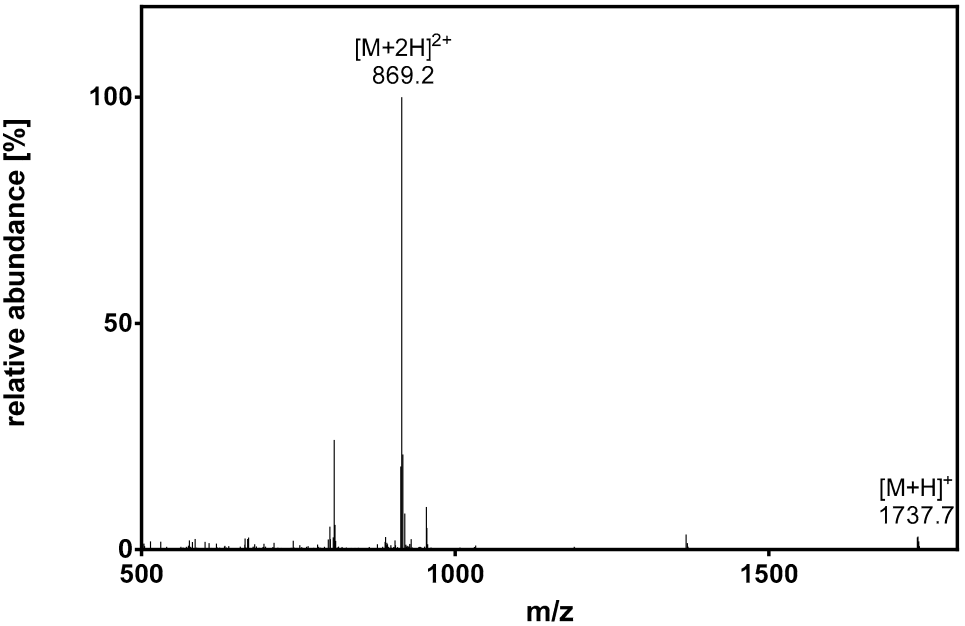

Figure S38. ESI-MS spectrum [C^{10}(chol)]PN9scram. ESI-MS (m/z): $[M + 2H]^{2+}$ 869.2 (found), 869.2 (calcd).

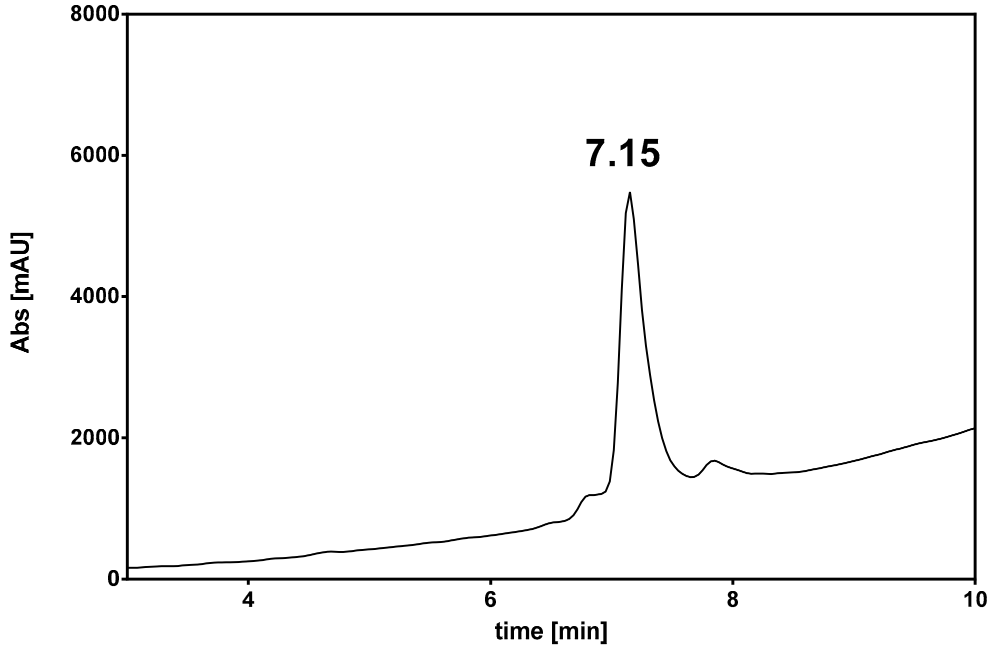

Figure S39. RP-HPLC traces of the peptide [C^{10}(chol)]PN9scramble-spacer-PEG_6. Conditions: C4 column Kromasil (5 µm × 4.6 × 150 mm); temperature, 308 K; flow, 1 mL/min; eluents, 0.1% (*v*/*v*) TFA in H_2O (A) and 0.1% (*v*/*v*) TFA in ACN (B); λ, 215 nm; gradient, 50 - 100% B in A over 10 min. R_t = 7.15 min.

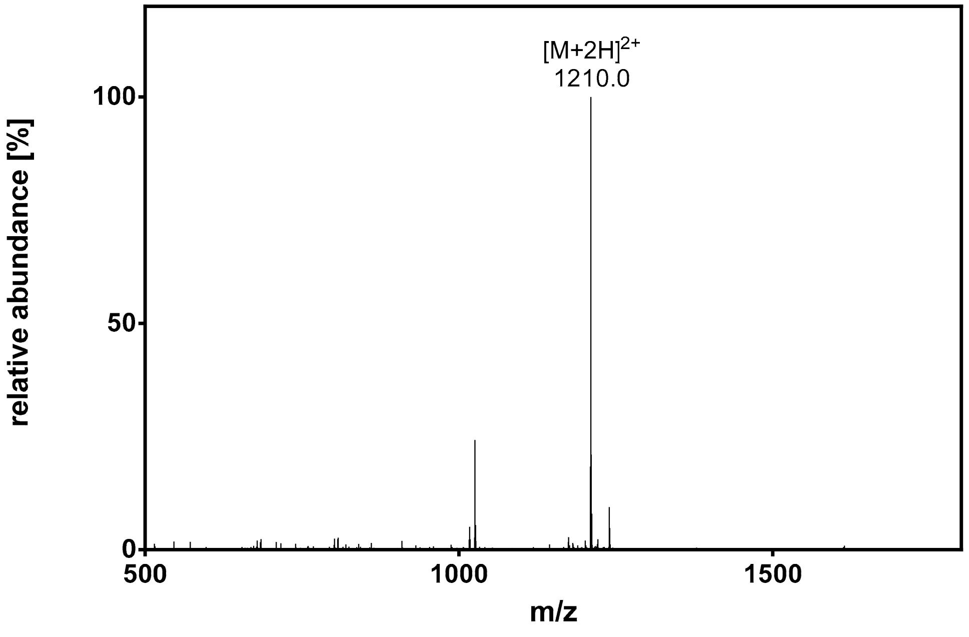

Figure S40. ESI-MS spectrum [C^{15}(chol)]PN9scramble-spacer-PEG_6. ESI-MS (m/z): $[M + 2H]^{2+}$ 1210.0 (found), 1209.7 (calcd).

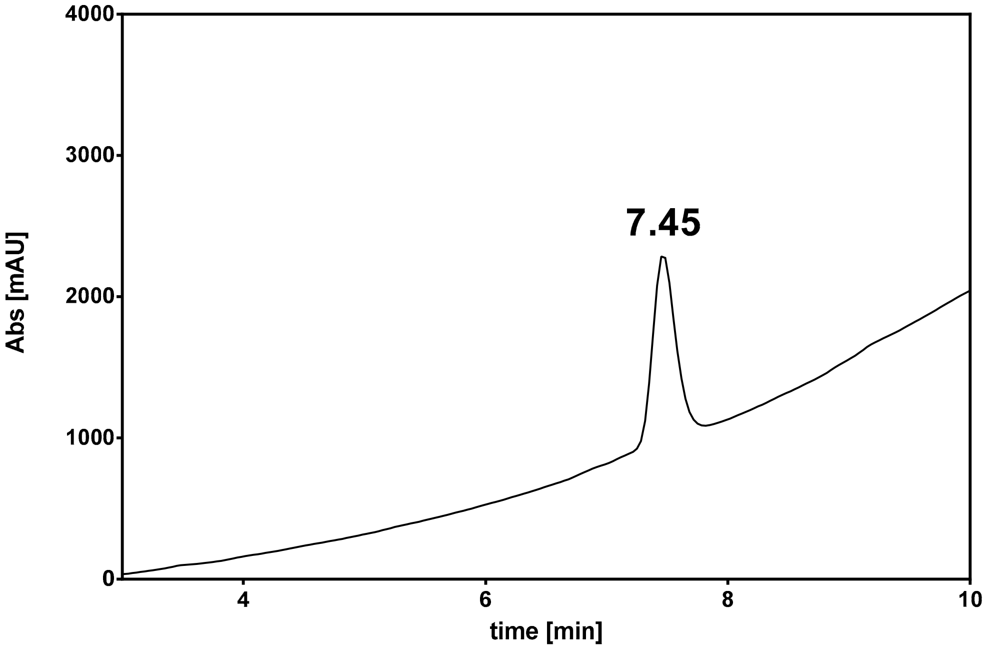

Figure S41. RP-HPLC traces of the peptide [C^{15}(chol)]PN9-spacer-PEG$_{12}$. Conditions: C4 column Kromasil (5 µm × 4.6 × 150 mm); temperature, 308 K; flow, 1 mL/min; eluents, 0.1% (*v*/*v*) TFA in H_2O (A) and 0.1% (*v*/*v*) TFA in ACN (B); λ, 215 nm; gradient, 50 - 100% B in A over 10 min. R_t = 7.45 min.

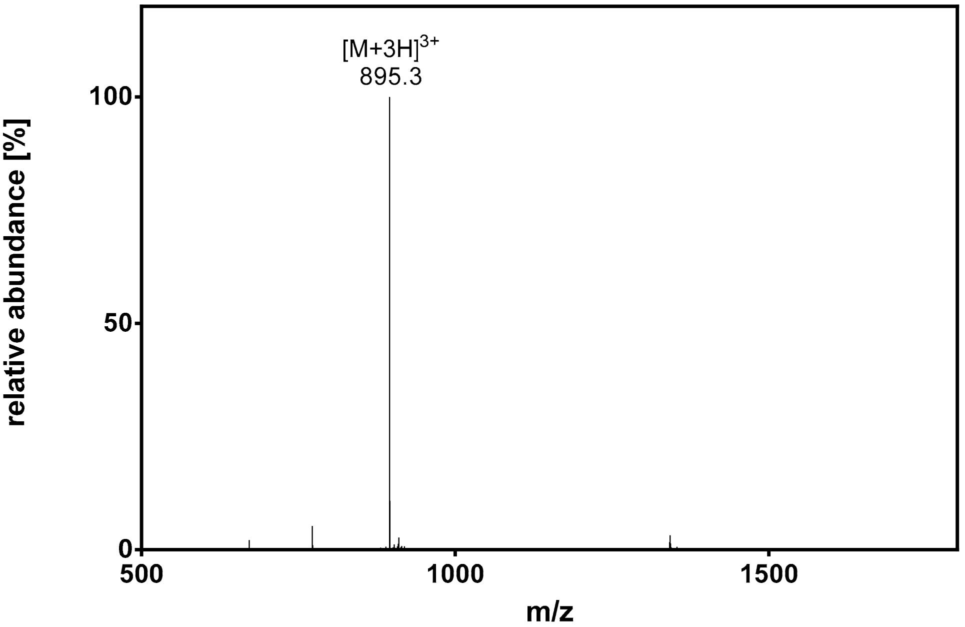

Figure S42. ESI-MS spectrum [C^{15}(chol)]PN9-spacer-PEG$_{12}$. ESI-MS (m/z): [M + 3H]$^{3+}$ 895.3 (found), 895.1 (calcd).
